# Comparative phenotypic and genotypic analysis of distinct *Pseudomonas aeruginosa* T3SS effector genotypes

**DOI:** 10.3389/fcimb.2026.1792519

**Published:** 2026-04-15

**Authors:** Nada K. Alharbi, Naser A. El-Sawy, Naief A. Dahran, Banan Atwah, Abdullah G. Al-Kushi, Maha Alharbi, Sozan M. Abdelkhalig, Rasha A. Mosbah, Abdallah Tageldein Mansour, Mahmoud M. Bendary

**Affiliations:** 1 Department of Biology, College of Science, Princess Nourah bint Abdulrahman University, Riyadh, Saudi Arabia; 2 Department of Anatomy and Embryology, Faculty of medicine, Zagazig university, Zagazig, Egypt; 3 Department of Basic Medical Sciences, College of Medicine, University of Jeddah, Jeddah, Saudi Arabia; 4 Department of Clinical Laboratory Sciences, College of Applied Medical Sciences, Umm Al-Qura University, Makkah, Saudi Arabia; 5 Department of Anatomy, Faculty of Medicine, Umm Al-Qura University, Makkah, Saudi Arabia; 6 Department of Basic Medical Sciences, College of Medicine, AlMaarefa University, Diriyah, Riyadh, Saudi Arabia; 7 Research Center, Deanship of Scientific Research and Post-Graduate Studies, AlMaarefa University, Diriyah, Riyadh, Saudi Arabia; 8 Infection Control Unit, Zagazig University Hospital, Zagazig, Egypt; 9 Animal and Fish Production, College of Agricultural and Food Sciences, King Faisal University, Al-Ahsa, Saudi Arabia; 10 Department of Microbiology and Immunology, Faculty of Pharmacy, Port Said University, Port Said, Egypt

**Keywords:** antimicrobial resistance, disease severity, exoU+, *P. aeruginosa*, T3SS effectors, virulence

## Abstract

**Background:**

*Pseudomonas aeruginosa* (*P. aeruginosa*) causes healthcare- and community-associated infections with high antimicrobial resistance and virulence diversity. The type III secretion system (T3SS) effectors genotypes (*exoS+, exoU+, exoT+, and exoY+*) play central roles in pathogenesis, yet their relationships with antimicrobial resistance, virulence profiles, serotypes, and clinical manifestations remain incompletely defined.

**Methods:**

Five hundred clinical specimens, sputum, urine, burn, wound, and eye exudates, 100 each, were collected from multiple hospitals for isolation of P. aeruginosa. Phenotypic and genotypic methods identified isolates and characterized T3SS effector genotypes, antimicrobial susceptibility, resistance genes, virulence genes, and serotypes. Clustering, strain typing, and correlation analyses assessed associations among resistance, virulence, epidemiology, and disease severity.

**Results:**

Among 125 isolates, exoS+ was the most prevalent T3SS effector, followed by exoU+, while exoT+ and exoY+ were less frequent. The exoU+ isolates were consistently associated with severe clinical manifestations across specimen types and were more often hospital acquired, with enrichment for serotypes O6 and O11. The exoS+ isolates showed broader serotype diversity with balanced hospital and community acquisition, whereas ExoT and ExoY were mainly associated with O6 and O11, with ExoY more common in community-acquired cases but less prevalent overall. Despite widespread antimicrobial resistance and broad distribution of resistance genes, polymyxin remained the most effective agent, with a low resistance rate of 15.2%. Multidrug resistance affected 81.6% of isolates and was observed across all T3SS effector genotypes, with frequent coexistence of resistance and virulence genes within individual isolates. Hierarchical clustering revealed marked intra-group and inter-group heterogeneity, with no evidence of dominant clonal expansion by effector genotype or specimen type, and correlation analysis showed weak associations among resistance, virulence, serotypes, and epidemiological features, except for a consistent link between exoU+ and markers of severe tissue injury.

**Conclusions:**

*P. aeruginosa* isolates form a heterogeneous, non-clonal population with independent resistance and virulence traits, while T3SS effector genotypes, especially exoU+, best predicts disease severity. The data suggest that pathogenicity in *P. aeruginosa* is driven by independently assorting resistance and virulence traits, not by fixed clonal backgrounds. These findings support integrating T3SS effector profiling into clinical risk assessment and surveillance.

## Introduction

Infectious diseases represent a global health hazard because they cause high rates of illness and death, spread rapidly across borders through travel and trade, overwhelm healthcare systems, disrupt routine medical services, and increase economic and social burdens, especially in vulnerable populations (Ghaly et al., 2021; Bendary et al., 2016). *Pseudomonas aeruginosa* (*P. aeruginosa*) is a metabolically versatile Gram-negative bacterium that occurs naturally in water, soil, and moist environments and readily adapts to healthcare settings, where it is a major cause of opportunistic infections (Horikian et al., 2024). Its large genome and flexible metabolism enable survival under nutrient-poor and stressful conditions, while its tolerance to disinfectants and ability to form biofilms support long-term persistence in hospital water systems, sinks, ventilators, catheters, and other medical equipment (Ambreetha et al., 2024). Transmission occurs mainly through contact with contaminated surfaces, fluids, and the hands of healthcare workers, with infection risk markedly increased in patients with burns, indwelling devices, mechanical ventilation, prolonged hospitalization, or compromised immunity (Vincent et al., 2020). Clinically, *P. aeruginosa* is responsible for a wide spectrum of infections including ventilator-associated pneumonia, bloodstream infections, urinary tract infections, wound and burn infections, and ocular disease, often associated with high morbidity and mortality. Treatment is particularly challenging because the organism possesses intrinsic resistance to many antimicrobial classes and can rapidly acquire additional resistance through mutation and horizontal gene transfer, frequently leading to multidrug-resistant and extensively drug-resistant phenotypes (Schwartz et al., 2024). Together, its environmental persistence, efficient transmission in healthcare settings, virulence potential, and high resistance capacity have established *P. aeruginosa* as one of the most problematic pathogens in hospital-acquired infections and a persistent global public health threat.

*P. aeruginosa* is one of the most problematic bacterial pathogens due to its high level of intrinsic and acquired antimicrobial resistance, which severely limits therapeutic options and complicates clinical management (Pang et al., 2019). This organism possesses multiple intrinsic resistance mechanisms, including low outer-membrane permeability, multidrug efflux pumps and inducible chromosomal AmpC β-lactamase, which together reduce susceptibility to many β-lactams, fluoroquinolones, and aminoglycosides (Oliver et al., 2024). In addition, *P. aeruginosa* frequently acquires extended-spectrum β-lactamase (ESBL) genes such as *bla*_CTX-M_, *bla*_SHV_, and *bla*_TEM_, carbapenemase genes including *bla*_NDM_, *bla*_KPC_, and *_bla_*_OXA-48_, and aminoglycoside resistance genes such as 16S rRNA methylase genes rmtA, rmtB, and rmtF, leading to multidrug-resistant (MDR) and extensively drug-resistant (XDR) phenotypes (Horna et al., 2019). Resistance to fluoroquinolones commonly arises through chromosomal mutations in *gyrA, gyrB, parC*, and *parE*, as well as regulatory mutations affecting efflux systems, while resistance to polymyxins such as colistin and polymyxin B is increasingly linked to modifications of lipid A mediated by *pmrA/pmrB, phoP/phoQ* (Pang et al., 2019; Poirel et al., 2017). Clinically, resistance is most frequently reported against commonly used antipseudomonal agents including piperacillin-tazobactam (TZP), ceftazidime (CAZ), cefepime (FEP), aztreonam (ATM), imipenem (IMP), meropenem (MEM), ciprofloxacin (CIP), levofloxacin (LEV), gentamicin (GEN), amikacin (AMK), and tobramycin (TOB), while polymyxin B and colistin often remain last-line options despite emerging resistance (Bassetti et al., 2018). The frequent coexistence of multiple resistance genes within single isolates reflects strong antibiotic selective pressure in healthcare settings and highlights the organism’s remarkable capacity for adaptation through mutation and horizontal gene transfer. Collectively, these features explain why *P. aeruginosa* remains a leading cause of difficult-to-treat infections worldwide and underscore the urgent need for antimicrobial stewardship, continuous surveillance, and development of alternative therapeutic strategies (Lister et al., 2009; Pang et al., 2019).

*P. aeruginosa* shows extensive genetic diversity. Several genotyping approaches are used to investigate this diversity. Multilocus sequence typing (MLST) remains a widely used framework for studying population structure and global epidemiology because it analyzes conserved housekeeping genes and assigns isolates to defined sequence types. This method supports the identification of clonal relationships and high-risk lineages circulating in clinical settings (Flores-Vega et al., 2024). In contrast, Type III secretion system (T3SS) effector profiling does not aim to resolve phylogenetic relationships but rather examines the distribution of key virulence determinants such as *exoS*, *exoU*, *exoT*, and *exoY*. These effectors define functional pathogenic strategies and influence host–pathogen interaction, clinical severity, and resistance patterns (Hauser, 2009). In particular, the presence of *exoU* has frequently been associated with increased cytotoxicity, severe clinical outcomes, and multidrug resistance among clinical isolates (Subedi et al., 2018). Therefore, while MLST provides a robust framework for evolutionary and epidemiological analysis, T3SS effector genotyping offers a complementary approach focused on virulence potential and clinical impact.

The type III secretion system (T3SS) of *P. aeruginosa* delivers four principal effector proteins (EXOS, EXOU, EXOT, and EXOY) that contribute differently to disease severity and clinical presentation. EXOS is a multifunctional effector with GTPase-activating and ADP-ribosyl transferase activities that disrupt host cell signaling and inhibit phagocytosis, facilitating bacterial invasion, dissemination, and long-term persistence; exoS+ strains are frequently linked to urinary tract, ocular, and chronic respiratory infections (Reuven et al., 2024). In contrast, EXOU is a highly potent phospholipase A2 cytotoxin that rapidly destroys host cell membranes, causing necrosis and severe inflammation, and is strongly associated with acute invasive infections such as pneumonia, bloodstream infections, and burn wound infections, as well as increased mortality (Qin et al., 2022). EXOT, which is structurally related to EXOS but less enzymatically active, interferes with cell adhesion, wound repair, and immune cell migration, contributing mainly to epithelial barrier disruption and localized inflammation rather than extensive tissue destruction (Lu et al., 2024). EXOY, an adenylate cyclase, increases intracellular cyclic nucleotide levels and promotes vascular leakage and edema, but its role is generally supportive, and it shows weak associations with specific infection sites or clinical outcomes (Deruelle et al., 2026). These effectors define distinct but overlapping pathogenic strategies, ranging from invasive persistence to acute cytotoxic injury, and their frequent co-distribution across clinical samples highlights the flexible and adaptable virulence architecture of *P. aeruginosa* rather than strict genotype–site specialization.

*P. aeruginosa* possesses an extensive and diverse set of virulence factors that enable colonization, immune evasion, tissue damage, and persistence across a wide range of clinical infections, making it one of the most versatile bacterial pathogens (Moursi et al., 2025). Central to its pathogenicity is the type III secretion system (T3SS), which injects effector proteins directly into host cells. In addition to T3SS effectors, *P. aeruginosa* produces numerous secreted toxins and enzymes, including exotoxin A (*toxA*), which inhibits host protein synthesis, phospholipases C (*plcH*, *plcN*) that damage cell membranes, and proteases such as LasB elastase (*lasB*) and alkaline protease (*aprA*) that degrade host tissues and immune components (Moradali et al., 2017). Quorum-sensing systems, mediated by *lasI*/*lasR*, *rhlI*/*rhlR*, and *pqsA*/*pqsR*, regulate coordinated expression of many virulence traits, including toxin production, motility, and biofilm formation (Miranda et al., 2022). Surface-associated factors such as flagellin (*fliC*), type IV pili (*pilA*, *pilB*), and lipopolysaccharide O-antigen biosynthesis genes facilitate adhesion, motility, and immune stimulation, while biofilm-associated genes including *algD*, *algU*, *pslA*, *pelA*, and *mucA* promote chronic infection and resistance to host defenses and antimicrobials (Pang et al., 2019). The frequent coexistence of multiple virulence genes within single isolates highlights the organism’s modular virulence architecture and explains its ability to cause both acute invasive disease and chronic persistent infections. This broad virulence repertoire illustrates the clinical severity, adaptability, and global importance of *P. aeruginosa* as a major opportunistic pathogen (Moradali et al., 2017).

This study was based on the hypothesis that antimicrobial resistance and virulence traits in *P. aeruginosa* are not randomly distributed but instead are influenced by selective pressures such as hospital exposure, intensity of antibiotic use, infection severity, and local environmental conditions. It further proposes that some virulence effector profiles are more likely to be linked to severe tissue damage, poor clinical outcomes, and multidrug resistance, while other profiles are associated with persistence or less severe infections. The primary aim of this work was therefore to comprehensively characterize the antimicrobial resistance patterns, resistance genes, virulence gene repertoire, T3SS effector genotypes, serotypes, and epidemiological features of clinical *P. aeruginosa* isolates from diverse sample types, and to evaluate how these factors interact across hospital- and community-acquired infections. By integrating phenotypic susceptibility testing, molecular detection of resistance and virulence genes, serotyping, and clustering and correlation analyses, the study sought to determine whether specific virulence profiles or clinical sources predict resistance burden and disease severity, or whether *P. aeruginosa* populations instead exhibit a heterogeneous, non-clonal structure driven by local selective pressures rather than lineage expansion.

## Methodology

### Sample collection and preliminary identification of *P. aeruginosa*

A total of 500 clinical specimens were collected from hospitalized patients with suspected *P. aeruginosa* infection, preferably before antibiotic administration, from multiple hospitals across Egypt between December 2024 and February 2025. The specimens included sputum, urine, wound swabs, eye swabs, and burn exudates, with 100 samples collected from each specimen type. All specimens were obtained using sterile collection protocols, placed immediately into sterile containers, and transported to the microbiology laboratory under aseptic conditions while maintained at appropriate temperatures. Samples were processed upon arrival. Each specimen was first inoculated into nutrient broth and incubated aerobically at 37 °C for 18 to 24 hours. After enrichment, a loopful of culture was streaked onto cetrimide agar for selective isolation of *P. aeruginosa*. Samples showing abundant, uniform colonies consistent with *P. aeruginosa* on cetrimide agar at a concentration of at least 10^6^ CFU were considered positive. Clinical manifestations were documented for each case by qualified specialists. Infections were classified as community acquired or hospital acquired according to the Centers for Disease Control and Prevention criteria. Community acquired infections were defined by positive cultures obtained at admission or within 48 hours of hospitalization in patients without recent healthcare exposure, whereas hospital acquired infections were defined by cultures that became positive 48 hours or more after admission following an initially negative result. Of note, hospital-acquired eye infection was defined as an ocular infection that developed 48 hours or more after hospital admission or following an ophthalmic medical procedure, in a patient with no evidence of eye infection at the time of admission. These infections may occur as a result of exposure to contaminated medical equipment, surgical procedures, contact lenses used in clinical settings, or cross-transmission of pathogens within healthcare facilities.

### Assessment of clinical findings

Clinical findings were evaluated for all patients from whom *P. aeruginosa* isolates recovered. Assessment was performed at the time of specimen collection and during routine clinical examination by attending physicians in the corresponding hospital departments. For respiratory isolates obtained from sputum samples, clinical findings were recorded based on physical examination, oxygen saturation monitoring, radiological imaging, and sputum characteristics. Parameters included drop in peripheral oxygen saturation, presence of heavy purulent sputum, pulmonary infiltration, necrotizing changes, and airway edema. Oxygen saturation was measured using pulse oximetry, while pulmonary infiltration and necrotizing changes were confirmed by chest imaging. For urinary isolates, clinical findings were determined through urine analysis, patient symptoms, and clinical examination. Pyuria and hematuria were identified by microscopic urine examination. Necrosis, severe pain, and edema were documented based on clinical assessment and patient-reported symptoms. For wound and burn exudate isolates, clinical evaluation included inspection of the affected area by surgical or burn care specialists. Findings such as purulent discharge, wound necrosis, edema, wound deterioration, and tissue damage were recorded based on visual examination and routine wound assessment protocols. For eye exudate isolates, ophthalmologic examination was conducted by qualified ophthalmologists. Clinical findings including purulent discharge, corneal ulceration, necrosis, and edema were assessed using slit-lamp examination and standard ophthalmic diagnostic procedures. Clinical findings were recorded as present or absent for each case. The frequency of each clinical manifestation was calculated as a percentage of the total number of isolates within each specimen category. These data were then correlated with the corresponding T3SS effector genotypes for descriptive analysis.

### Phenotypic and genotypic identification of *Pseudomonas aeruginosa*

Presumptive identification of *P. aeruginosa* was based on colony morphology on cetrimide agar and Gram staining, which confirmed Gram-negative rod-shaped bacteria. Oxidase testing was performed, and oxidase-positive isolates were selected for further identification in accordance with standard clinical microbiology procedures (Forbes et al., 2007). Phenotypic identification was performed using the API 20NE identification system (BioMérieux, France) following the manufacturer’s instructions. Briefly, fresh pure colonies were suspended in sterile saline to achieve the recommended turbidity. The bacterial suspension was used to inoculate the microtubes of the API 20NE strip, and specific cupules were overlaid with sterile mineral oil when required. The strips were incubated aerobically at 37 °C for 24–48 hours. After incubation, color reactions were recorded, and additional reagents were added to designated tests as specified by the manufacturer. Identification was obtained by converting biochemical reactions into a numerical profile and interpreting the results using the API database (BioMérieux).

Genotypic confirmation was carried out by polymerase chain reaction targeting species-specific genes of *P. aeruginosa*. Genomic DNA was extracted from overnight cultures using standard extraction methods. PCR amplification was performed using primers specific for the *oprL* gene (**Supplementary Table 1**), which are conserved outer membrane lipoprotein genes commonly used for molecular identification of *P. aeruginosa* (DeVos et al., 1997; Spilker et al., 2004). Amplified products were analyzed by agarose gel electrophoresis and visualized under ultraviolet illumination. Isolates producing amplicons of the expected size were confirmed as *P. aeruginosa*. Positive and negative controls were included in all PCR assays to ensure assay validity.

### T3SS effector genotypic characterization by multiplex PCR

Genotypic characterization of Type III Secretion System effector genes was performed in triplicate using multiplex polymerase chain reaction targeting *exoS*, *exoT*, *exoU*, and *exoY*. Genomic DNA was extracted from bacterial isolates using a standard bacterial DNA extraction procedure. Briefly, overnight bacterial cultures were centrifuged, and the cell pellet was resuspended in lysis buffer containing Tris-HCl, EDTA, and lysozyme. Cells were incubated to allow enzymatic lysis followed by protein digestion with proteinase K and sodium dodecyl sulfate. DNA was then purified by phenol–chloroform extraction or a commercial genomic DNA purification kit and precipitated with ethanol. The purified DNA was resuspended in nuclease-free water and quantified prior to amplification. Multiplex PCR was carried out using specific primer pairs for *exoS*, *exoT*, *exoU*, and *exoY* as previously described (**Supplementary Table 1**).

Each PCR reaction was prepared in a final volume of 25 µL containing genomic DNA template, primer mixtures, PCR buffer, MgCl_2_, dNTPs, and Taq DNA polymerase. The multiplex PCR program consisted of an initial denaturation at 94 °C for 3 minutes, followed by denaturation at 94 °C for 40 seconds, annealing at 58 °C for 40 seconds, and extension at 68 °C for 1 minute. The reaction was completed with a final extension step at 68 °C for 1 minute (Jarjees, 2020; Elnagar et al., 2022). Laboratory strains of *P. aeruginosa* maintained in our laboratory collection were used as positive controls for validation of the multiplex PCR assay. These strains had been previously characterized in our laboratory and were known to harbor one of the Type III secretion system effector genes (exoS, exoT, exoU, or exoY). Prior confirmation of these genes was established in triplicate through earlier PCR screening using gene-specific primers, and the presence of the corresponding amplicons was verified based on the expected fragment sizes following agarose gel electrophoresis. A negative control containing all PCR reaction components except template DNA was included in each PCR run to monitor contamination and nonspecific amplification. Amplified PCR products were separated by electrophoresis on a 1.5 percent agarose gel stained with intercalating dye and visualized under ultraviolet illumination.

The presence of bands at expected sizes was considered positive for the corresponding T3SS effector genes. Isolates were categorized into Type III secretion system effector gene profiles based on multiplex PCR detection of exoS+, exoT+, exoU+, and exoY+. Because *exoT* and *exoY* genes are commonly conserved among *P. aeruginosa* lineages we treated them as core effector markers and defined the main virulotype by the presence of *exoS* or *exoU*. Isolates were classified as *exoS* positive *exoU* negative or *exoU* positive *exoS* negative with concurrent reporting of *exoT* and *exoY* carriage in each group and any atypical combinations were recorded separately. However, isolates were classified as exoY+, exoT+, or exoY+/exoT+ genotypes when they carried the *exoY* gene only, the *exoT* gene only, or both genes, respectively.

### Antimicrobial susceptibility testing

Antimicrobial susceptibility testing of *P. aeruginosa* was performed using disk diffusion and broth microdilution methods according to standardized guidelines. A fresh isolate was adjusted to a 0.5 McFarland standard and inoculated onto Mueller–Hinton agar to obtain a uniform lawn, after which antibiotic disks were applied, and plates were incubated aerobically at 35–37 °C for 16–18 h. The antibiotics tested were grouped by class and included polymyxins, Polymyxin B (PMB); monobactams, Aztreonam (ATM); penicillins and penicillin/β-lactamase inhibitor combinations, Piperacillin (PIP) and Ticarcillin–clavulanate (TIC); cephalosporins and cephalosporin/β-lactamase inhibitor combinations, Cefiderocol (FDC), Ceftolozane–tazobactam (C/T), Ceftazidime (CAZ), and Cefepime (FEP); aminoglycosides, Gentamicin (GEN) and Amikacin (AMK); fluoroquinolones, Ciprofloxacin (CIP) and Levofloxacin (LVX); and carbapenems, Imipenem (IMP), Meropenem (MEM), and Doripenem (DOR). Zone diameters were measured in millimeters and interpreted using the relevant CLSI breakpoints (Clinical and Laboratory Standards Institute, 2024).

For broth microdilution, serial twofold dilutions of selected agents, including Polymyxin B, were prepared in cation-adjusted Mueller–Hinton broth, inoculated with a standardized bacterial suspension, and incubated at 35–37 °C for 16–20 h, after which the minimum inhibitory concentration was recorded as the lowest concentration preventing visible growth and interpreted according to guideline-recommended MIC breakpoints (Clinical and Laboratory Standards Institute, 2024). Multidrug resistance and extensive drug resistance were defined according to internationally accepted criteria. Multidrug-resistant (MDR) bacteria were defined as isolates showing non-susceptibility to at least one agent in three or more antimicrobial classes that are normally active against the organism. Extensively drug-resistant (XDR) bacteria were defined as isolates remaining susceptible to only one or two antimicrobial classes while showing non-susceptibility to all other tested classes (Magiorakos et al., 2012).

### Molecular characterization of resistance and other virulence genes

Genomic DNA was extracted from overnight cultures of *P. aeruginosa* using a commercial bacterial DNA extraction kit according to the manufacturer instructions. DNA concentration and purity were determined spectrophotometrically, and samples were stored at −20 °C until use. Molecular detection of virulence and biofilm associated genes *toxA, aprA, lasB, plcH, rhlAB, fliC, lasI, lasR, rhlI, rhlR, algD, pslA, pelA, pvdA, pilA*, and *pilB* was performed by conventional polymerase chain reaction using previously published primer sets (**Supplementary Table 1**). PCR reactions were carried out in a final volume of 25 µL containing 12.5 µL of 2× PCR master mix, 1 µL of each primer at 10 pmol, 2 µL of template DNA, and nuclease free water. Several laboratory strains of *P. aeruginosa* previously confirmed to harbor the investigated virulence genes were used as positive controls, while a negative control containing nuclease free water instead of DNA template was included in each PCR run (Sabharwal et al., 2014; Rezaloo et al., 2022; Wang et al., 2025).

For *toxA*, *lasB*, *plcH*, *rhlAB*, *lasI*, *lasR*, *rhlI*, and *rhlR*, PCR was performed with an initial denaturation at 95 °C for 5 min, followed by 35 cycles of denaturation at 95 °C for 30 s, annealing at 58 °C for 30 s, extension at 72 °C for 45 s, and a final extension at 72 °C for 7 min (Sabharwal et al., 2014); for *aprA*, amplification was carried out using an initial denaturation at 95 °C for 5 min, followed by 35 cycles of denaturation at 95 °C for 30 s, annealing at 60 °C for 30 s, extension at 72 °C for 90 s, and a final extension at 72 °C for 10 min (Sabharwal et al., 2014); for *fliC*, PCR conditions consisted of an initial denaturation at 95 °C for 5 min, followed by 35 cycles of denaturation at 95 °C for 30 s, annealing at 55 °C for 30 s, extension at 72 °C for 90 s, and a final extension at 72 °C for 10 min (Sabharwal et al., 2014); for *algD*, *pslA*, and *pelA*, PCR amplification was performed with an initial denaturation at 95 °C for 5 min, followed by 35 cycles of denaturation at 95 °C for 30 s, annealing at 60 °C for 30 s, extension at 72 °C for 45 s, and a final extension at 72 °C for 7 min (Wang et al., 2025); for *pvdA*, PCR was carried out using an initial denaturation at 95 °C for 5 min, followed by 35 cycles of denaturation at 95 °C for 30 s, annealing at 58 °C for 30 s, extension at 72 °C for 90 s, and a final extension at 72 °C for 10 min (Rezaloo et al., 2022; Fazeli and Momtaz, 2014); and for *pilA* and *pilB*, amplification conditions consisted of an initial denaturation at 95 °C for 5 min, followed by 35 cycles of denaturation at 95 °C for 30 s, annealing at 55 °C for 30 s, extension at 72 °C for 45 s, and a final extension at 72 °C for 7 min (Rezaloo et al., 2022). PCR products were analyzed by electrophoresis on 1.5 percent agarose gels and visualized under ultraviolet illumination.

In our study, antimicrobial resistance genes were analyzed by conventional PCR using published primer sets (**Supplementary Table 1**). Carbapenemase genes *bla*_NDM_, *bla*_KPC_, and *bla*_OXA-48_, ESBL genes *bla*_SHV_, *bla*_TEM_, and *bla*_CTX-M_ (Chia et al., 2005, Gurung et al., 2020, Mlynarcik et al., 2016, Mohanam and Menon, 2022, Nguyen et al., 2018), the 16S rRNA methylase genes *rmtA*, and chromosomal resistance loci *gyrA* and *pmrA* were amplified using gene-specific annealing temperatures ranging from 51 to 72 °C with a uniform extension step at 72 °C. Each 25 microliter PCR reaction contained Taq polymerase master mix, forward and reverse primers, template DNA, and nuclease-free water, and thermal cycling conditions included an initial denaturation at 94 °C for 5 minutes, followed by 35 cycles of denaturation, annealing, and extension, and a final extension at 72 °C for 7 minutes. Amplified products were resolved on 1.5 percent agarose gel, visualized under UV transillumination, and interpreted based on expected amplicon sizes using a 100 bp DNA ladder, with positive and negative controls included in each run to ensure assay reliability.

### Serotyping

Serotyping of *P. aeruginosa* isolates was performed by slide agglutination based on O-antigen specificity. Fresh overnight colonies were suspended in sterile physiological saline on a clean glass slide to obtain a smooth homogenous suspension, after which an equal volume of type-specific O antisera was added. The mixture was gently rocked for up to 60 s and observed for visible agglutination under adequate illumination. A positive reaction was defined by the appearance of distinct clumping compared with the saline control. Each isolate was tested sequentially against commercially available O antisera panels covering the most prevalent *P. aeruginosa* serogroups, following previously described methods (Kusama, 1978; Mikkelsen, 1970; Holder et al., 1995). Reference *P. aeruginosa* ATCC 15692 for serotyping O5was used as positive controls, while a saline suspension without antiserum served as the negative control to verify assay specificity.

### Statistical analysis

Data analysis was performed using the R statistical environment (R – program, version 4.x). All quantitative variables were assessed for normality and scaled prior to analysis. Relationships among clinical characteristics, virulence profiles, and antimicrobial resistance patterns were examined using correlation analysis. Pearson correlation coefficients were calculated for variables meeting parametric assumptions. Correlation values were used to describe the direction and strength of associations between variables. Correlation matrices were constructed and visualized using appropriate R packages to illustrate pairwise relationships. Correlation coefficients (*r-value*) were assessed for both direct and inverse relationships. The strength of association was classified based on the absolute value of the correlation coefficient, with values below 0.3 considered weak, values between 0.3 and 0.7 considered moderate, and values of 0.7 or higher considered strong. Standardized binary data representing the presence or absence of clinical, virulence, and resistance features were further analyzed using heat map visualization. Hierarchical clustering was performed on both isolates and variables using Euclidean distance and complete linkage methods. Missing data were handled through imputation based on central tendency measures, and results were verified through repeated analyses to ensure robustness.

## Results

### Distribution of T3SS effector genotypes and their association with sample origin and clinical severity in *P. aeruginosa* isolates

**Figure 1:**
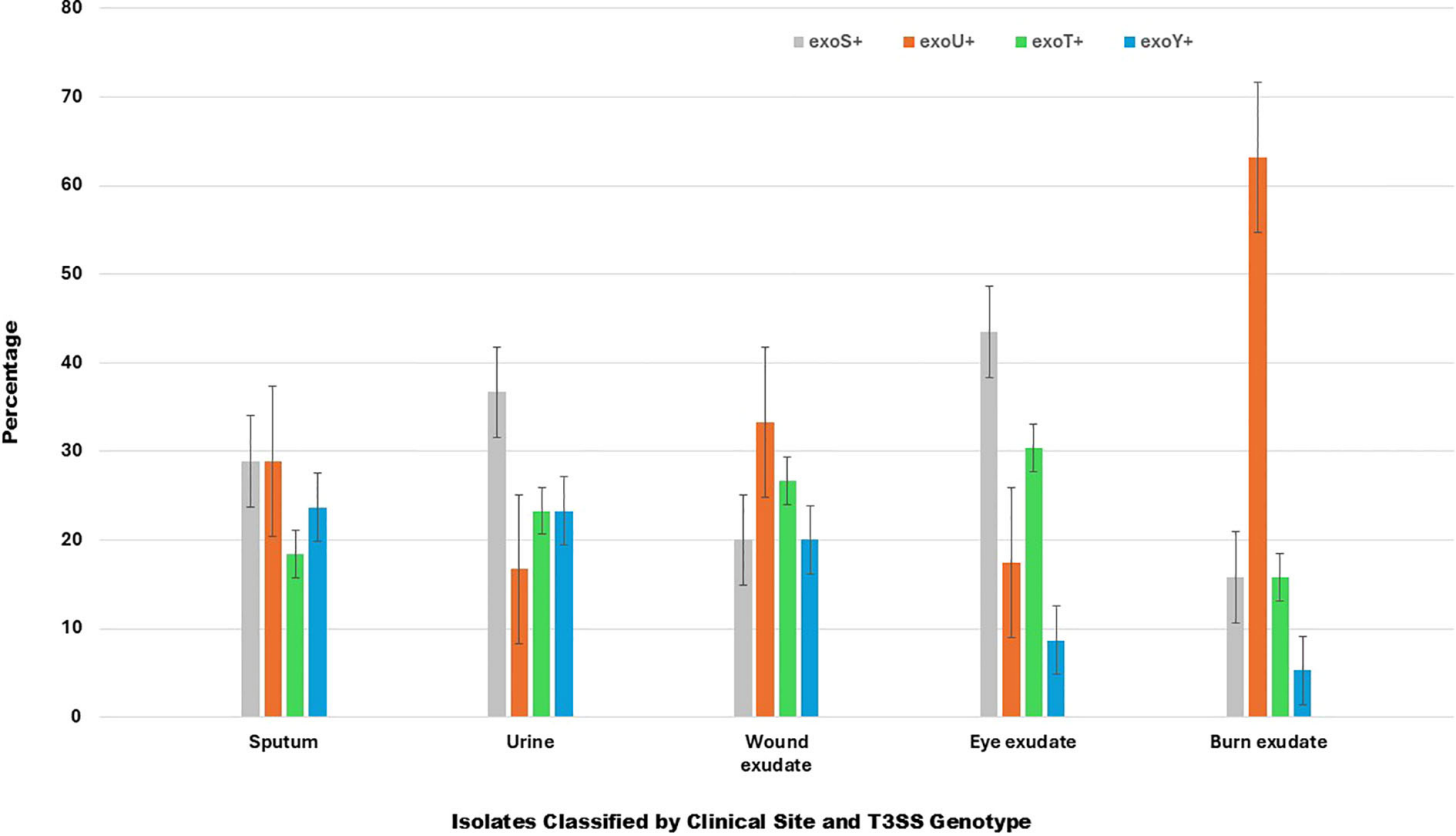
Percentage of *Pseudomonas aeruginosa* genotypes (exoS+_, exoU+, exoT+, and exoY+) across different clinical specimens (sputum, urine, wound, eye, and burn exudates). Error bars indicate standard deviation. Percentages were calculated based on the total number of isolates obtained from each clinical specimen.

A total of 125 P*. aeruginosa* isolates were confirmed from 500 clinical specimens of different types, with an overall prevalence of 25%. The exoS+ genotype was the most prevalent, accounting for 38 isolates (30.4%), while the exoU+ genotype comprised 37 isolates (29.6%). Both genotypes showed an equal distribution across sputum specimens (28.9% each). However, the exoS+ genotype was more prevalent in urine (36.7%) and eye exudates (43.5%). In contrast, the exoU+ genotype was more frequently detected in burn exudates (63.2% of isolates) (**Table 1**; **Figure 1**). The exoT+ genotype was identified in 28 cases (22.4%) and was most commonly detected in eye exudates (30.4%), whereas the exoY+ genotype was the least frequent, with 22 isolates (17.6%), and was most commonly detected in urine and sputum samples (23.7% each). Regarding sample types, sputum was the most common source with 38 isolates (30.4%), followed by urine with 30 isolates (24.0%), reflecting the high burden of respiratory and urinary tract infections. Eye exudate accounted for 23 isolates (18.4%), while burn exudate contributed 19 isolates (15.2%), both representing significant reservoirs of infection in tissue-compromised settings. Wound exudate was the least frequent source with 15 isolates (12.0%) (**Table 1**; **Figure 1**). This distribution indicates that lower respiratory and urinary specimens together constituted more than half of all isolates, underscoring their dominant role in clinical *Pseudomonas* infections.

**Table 1:** Distribution of *Pseudomonas aeruginosa* isolates from different clinical samples based on exoS+, exoU+, exoT+, and exoY+ genotypes and associated clinical findings.

Type of isolates [no]	Genotypes [no/total, (%)]				Clinical findings (%)				
	exoS+ 38	exoU+ 37	exoT+ 28	exoY+ 22					
Sputum (Magiorakos et al., 2012)	11/38 (28.9)	11/38 (28.9)	7/38 (18.4)	9/38 23.7)	Drop in SpO_2_	Heavy purulent sputum	Pulmonary infiltration	necrotizing changes	Airway edema
					65.8	57.9	34.2	28.9	15.8
Urine (Hueck, 1998)	11/30 (36.7)	5/30 (16.7)	7/30 (23.3)	7/30 (23.3)	Pyuria	Hematuria	Necrosis	Sever pain	Edema
					63.3	13.3	26.7	53.3	16.7
Wound exudate (Elnagar et al., 2022)	3/15 (20)	5/15 (33.3)	4/15 (26.7)	3/15 (20)	Purulent discharge	Wound necrosis	Edema	Wound deterioration	
					46.7	33.3	20	33.3	
Eye exudate (Hardy et al., 2022)	10/23 (43.5)	4/23 (17.4)	7/23 (30.4)	2/23 (8.7)	Purulent discharge	Corneal ulcer	Necrosis	Edema	
					65.2	13	13	13	
Burn exudate (Foulkes et al., 2019)	3/19 (15.8)	12/19 (63.2)	3/19 (15.8)	1/19 (5.3)	Purulent discharge	Necrosis	Edema		
	68.4				36.8	36.8			

Clinical manifestations varied markedly by sample site and showed a strong, non-random association with effector genotype. The exoU+ genotype consistently clustered with severe pathological features across anatomical sites. Of note, burn isolates showed a sharp contrast between exoU+ and non-exoU+ genotypes. The exoU+ isolates exhibited purulent discharge in 90.9% of cases and both necrosis and edema in 63.6%. In contrast, the total burn population showed purulent discharge in only 15.8%, with no necrosis or edema detected outside exoU+. This indicates that all necrotic and edematous burn pathology was confined to exoU+ isolates. Despite the small number of exoU+ strains among the eye infections, exoU+ was associated with destructive ocular findings including corneal ulceration and necrosis. This genotype was associated primarily with purulent discharge at 75%, while corneal ulceration and necrosis each occurred in 25% of cases. Edema was absent among exoU+ eye isolates. At the site level, purulent discharge was common at 52.2%, whereas corneal ulceration and necrosis were infrequent at 8.7% each, and edema occurred in 13%. These data show that exoU+ increases destructive ocular findings but does not account for all inflammatory changes (**Table 1**; **Supplementary Table 2**).

Regarding respiratory isolates, they displayed the strongest separation between effector genotype and clinical severity. All exoU+ sputum isolates demonstrated necrotizing changes at 100%, with hypoxemia and heavy purulent sputum each present in 81.8%. Pulmonary infiltration was uncommon at 9.1%, and airway edema was absent. In contrast, at the site level, necrotizing changes were not observed at all, while hypoxemia, heavy purulence, and pulmonary infiltration occurred at 42.1%, 34.2%, and 31.6%, respectively. This confirms that necrotizing lung pathology was exclusive to exoU+. Urinary isolates showed profound differences in symptom severity. The exoU+ isolates were uniformly associated with pyuria and severe pain at 100%, with hematuria and necrosis each present in 80% and edema in 40%. In comparison, total urine isolates showed pyuria in 46.7%, severe pain in 36.7%, necrosis in 13.3%, and edema in 10%, while hematuria was absent. Thus, nearly all severe urinary manifestations were clustered within exoU+ cases. Moreover, wound isolates again demonstrated exoU+ linked severity. The exoU+ isolates showed purulent discharge and wound necrosis in 80% of cases, edema in 60%, and wound deterioration in 80%. At the site level, purulent discharge occurred in only 20%, wound necrosis in 6.7%, wound deterioration in 6.7%, and edema was not detected. These findings indicate that advanced wound destruction was almost entirely restricted to exoU+ isolates (**Table 1**; **Supplementary Table 2**).

Across all sample types, exoU+ was consistently associated with necrosis and multi-feature clinical severity, while non-exoU+ isolates were predominantly linked to isolated inflammatory findings. This site-specific comparison indicates that effector genotype is a stronger determinant of tissue damage than anatomical location (**Table 1**; **Supplementary Table 2**).

### Antimicrobial resistance patterns

**Figure 2:**
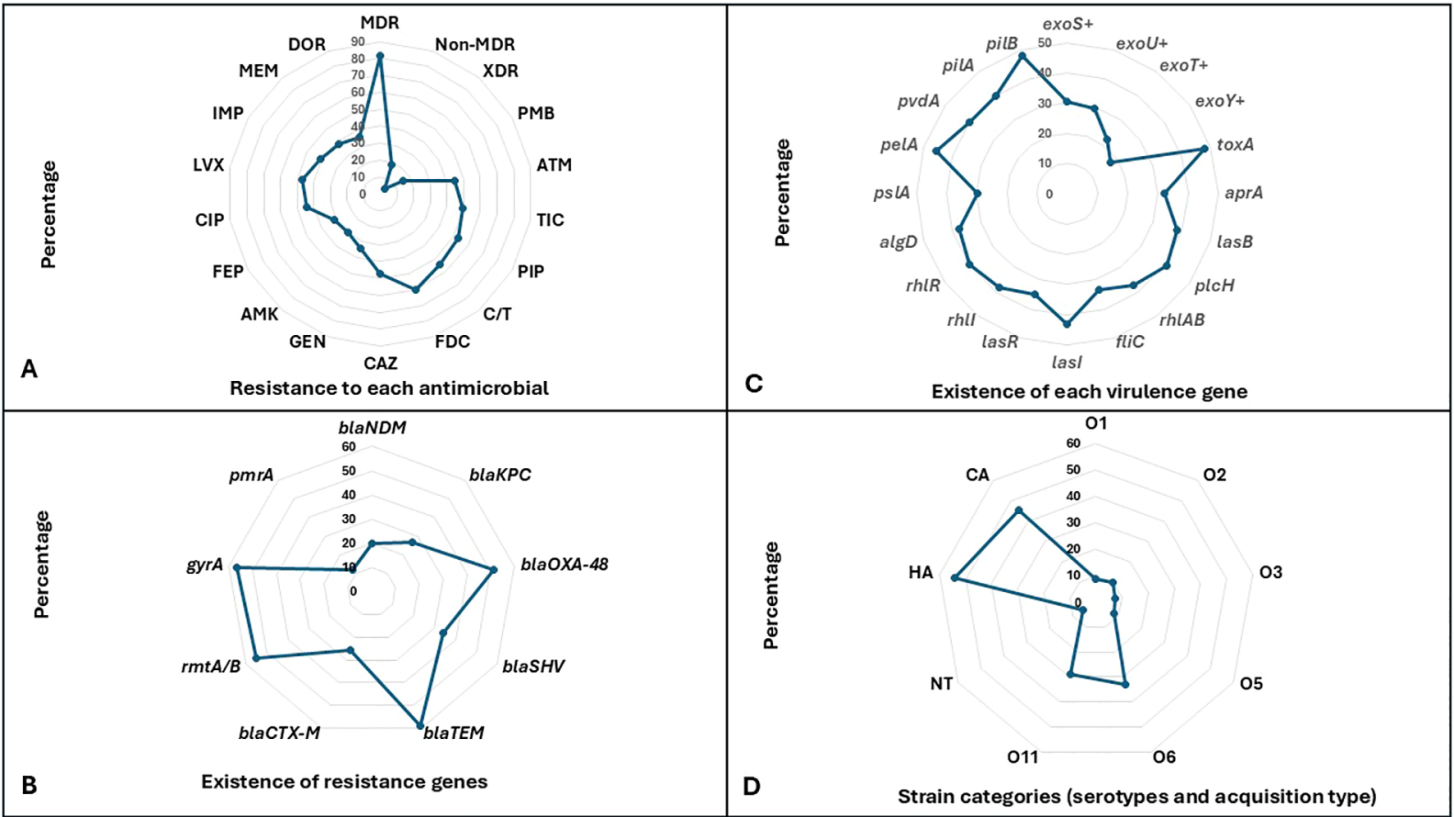
Distribution of antimicrobial resistance, virulence traits, and serotypes in *Pseudomonas aeruginosa* isolates. **(A)** presents resistance to each antimicrobial and resistance category, including non-MDR (non-multidrug resistant), MDR (multidrug resistant), XDR (extensively drug resistant), ATM (aztreonam), TIC (ticarcillin–clavulanate), PIP (piperacillin), C/T (ceftolozane–tazobactam), DOR (doripenem), CAZ (ceftazidime), FDC (cefiderocol), GEN (gentamicin), AMK (amikacin), FEP (cefepime), CIP (ciprofloxacin), LVX (levofloxacin), IMP (imipenem), and MEM (meropenem). **(B)** shows the prevalence of resistance genes, including *bla*_NDM_ (New Delhi metallo-β-lactamase), *bla*_KPC_ (Klebsiella pneumoniae carbapenemase), *bla*_OXA-48_ (oxacillinase-48), *bla*_SHV_ (sulfhydryl variable β-lactamase), *bla*_TEM_ (Temoneira β-lactamase), *bla*_CTX-M_ (cefotaximase), *rmtA* (16S rRNA methyltransferases A), *gyrA* (DNA gyrase subunit A mutation), and *pmrA* (polymyxin resistance regulator). **(C)** shows virulence gene distribution, including *exoS, exoU, exoT*, and *exoY* (type III secretion system effectors), *toxA* (exotoxin A), *aprA* (alkaline protease), *lasB* (elastase), *plcH* (hemolytic phospholipase C), *rhlAB* (rhamnolipid biosynthesis genes), *fliC* (flagellin), *lasI* and *lasR* (quorum sensing regulators), *rhlI* and *rhlR* (quorum sensing regulators), *algD* (alginate biosynthesis), *pslA* and *pelA* (biofilm formation genes), *pvdA* (pyoverdine synthesis), and *pilA* and *pilB* (type IV pili genes). **(D)** shows serotype distribution based on O-antigen, including O1, O2, O3, O5, O6, O11, NT (non-typeable strains) CA (community acquired isolates), HA (Hospital acquired isolates).

Antimicrobial susceptibility analysis identified multidrug resistance (MDR) in 81.6% of *P. aeruginosa* isolates, while 18.4% remained non-MDR. However, the Extensively drug-resistant (XDR) phenotypes accounted for 4% of the isolates. High resistance rates were observed against several antipseudomonal agents, particularly β-lactams and fluoroquinolones. Resistance to third-generation cephalosporins was pronounced, reaching 60% for Cefiderocol, 54.4% for Ceftolozane/Tazobactam, and 47.2% for ceftazidime, while resistance to piperacillin and Ticarcillin/clavulanate was also high at 52.8% and 49.6%, respectively. Fluoroquinolone resistance was similarly elevated, with non-susceptibility rates of 44% for ciprofloxacin and 47.2% for levofloxacin. Moderate resistance was observed for carbapenems, including imipenem at 40.8%, meropenem at 38.4%, and Doripenem at 36%. In contrast, lower resistance rates were recorded for amikacin at 29.6% and polymyxin B at 15.2%, indicating partial preservation of activity for these agents (**Figure 2A**).

**Figure 3:**
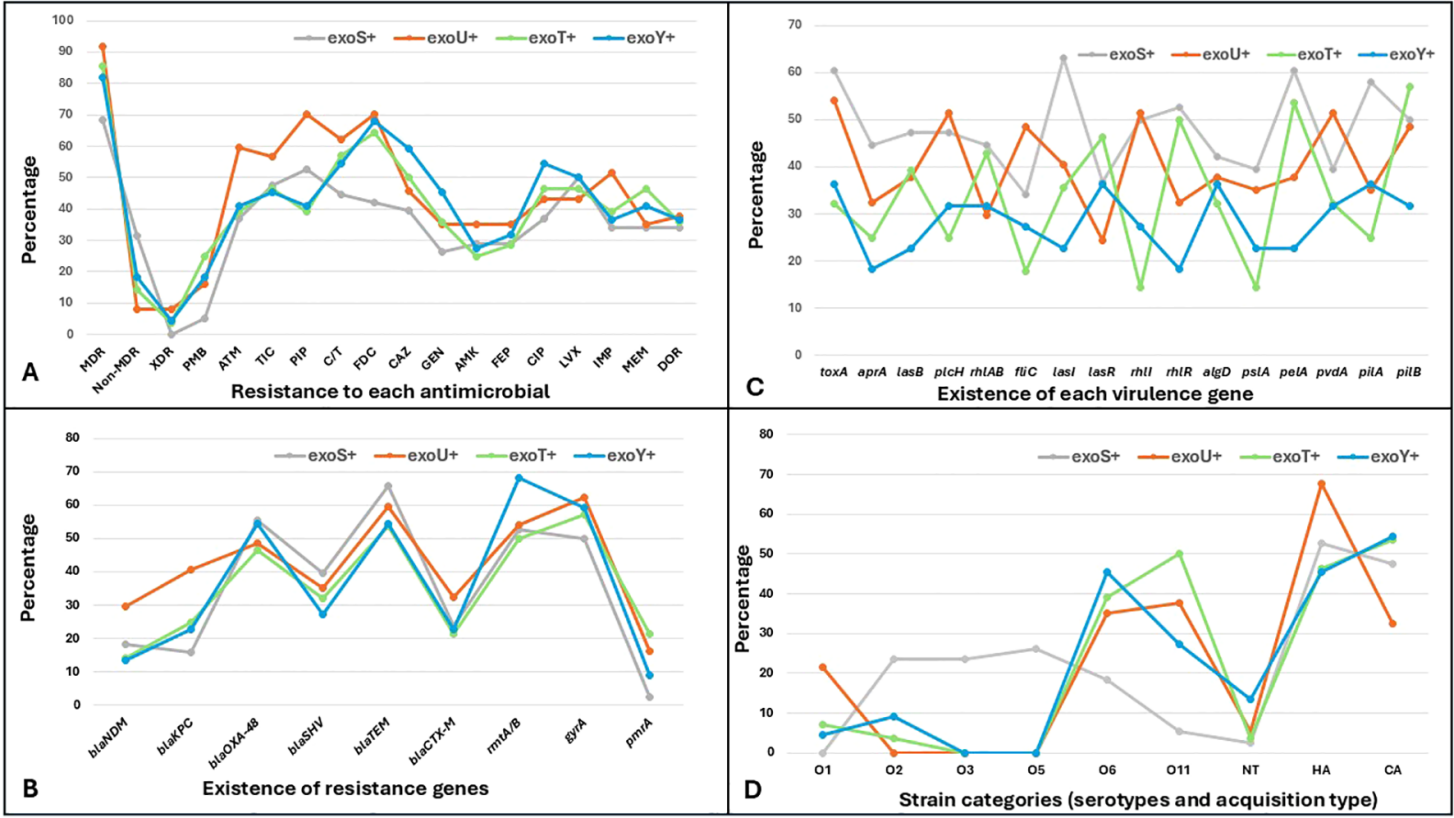
Comparative genomic and phenotypic profiles of *Pseudomonas aeruginosa* isolates stratified by type III secretion system effector genotypes. Isolates are grouped according to type III secretion system effector genotypes exoS+, exoU+, exoT+, and exoY+. **(A)** presents resistance to each antimicrobial and resistance category, including non-MDR (non-multidrug resistant), MDR (multidrug resistant), XDR (extensively drug resistant), ATM (aztreonam), TIC (ticarcillin–clavulanate), PIP (piperacillin), C/T (ceftolozane–tazobactam), DOR (doripenem), CAZ (ceftazidime), FDC (cefiderocol), GEN (gentamicin), AMK (amikacin), FEP (cefepime), CIP (ciprofloxacin), LVX (levofloxacin), IMP (imipenem), and MEM (meropenem). **(B)** shows the prevalence of resistance genes, including *bla*_NDM_ (New Delhi metallo-β-lactamase), *bla*_KPC_ (Klebsiella pneumoniae carbapenemase), *bla*_OXA-48_ (oxacillinase-48), *bla*_SHV_ (sulfhydryl variable β-lactamase), *bla*_TEM_ (Temoneira β-lactamase), *bla*_CTX-M_ (cefotaximase), *rmtA* (16S rRNA methyltransferases A), *gyrA* (DNA gyrase subunit A mutation), and *pmrA* (polymyxin resistance regulator). **(C)** shows virulence gene distribution, including *exoS, exoU, exoT*, and *exoY* (type III secretion system effectors), *toxA* (exotoxin A), *aprA* (alkaline protease), *lasB* (elastase), *plcH* (hemolytic phospholipase C), *rhlAB* (rhamnolipid biosynthesis genes), *fliC* (flagellin), *lasI* and *lasR* (quorum sensing regulators), *rhlI* and *rhlR* (quorum sensing regulators), *algD* (alginate biosynthesis), *pslA* and *pelA* (biofilm formation genes), *pvdA* (pyoverdine synthesis), and *pilA* and *pilB* (type IV pili genes). **(D)** shows serotype distribution based on O-antigen, including O1, O2, O3, O5, O6, O11, NT (non-typeable strains) CA (community acquired isolates), HA (Hospital acquired isolates).

Comparison of antimicrobial resistance across T3SS genotypes revealed clear differences, although resistance patterns overlapped substantially. The exoU+ isolates exhibited the highest resistance levels across most antimicrobial classes, particularly β-lactams and carbapenems. Resistance among exoU+ isolates reached 70.3% for piperacillin and Cefiderocol, 62.2% for Ceftolozane/Tazobactam, and 51.4% for imipenem, with fluoroquinolone resistance also elevated at 43.2% for both ciprofloxacin and levofloxacin. The exoS+ isolates demonstrated moderate resistance across multiple agents, including piperacillin at 52.6%, Ticarcillin/clavulanate at 47.4%, and levofloxacin at 50%, but generally remained lower than exoU+ for cephalosporins and carbapenems. The exoT+ and exoY+ isolates showed comparable or higher resistance to selected cephalosporins, particularly Cefiderocol at 64.3% and 68.2% and ceftazidime at 50% and 59.1%, respectively, but displayed lower resistance to carbapenems and polymyxin B. While certain resistance profiles were associated with genotypes, the extensive overlap in resistance frequencies across all groups suggesting heterogeneous resistance profiles instead of genotype-specific patterns (**Figure 3A**).

**Figure 4:**
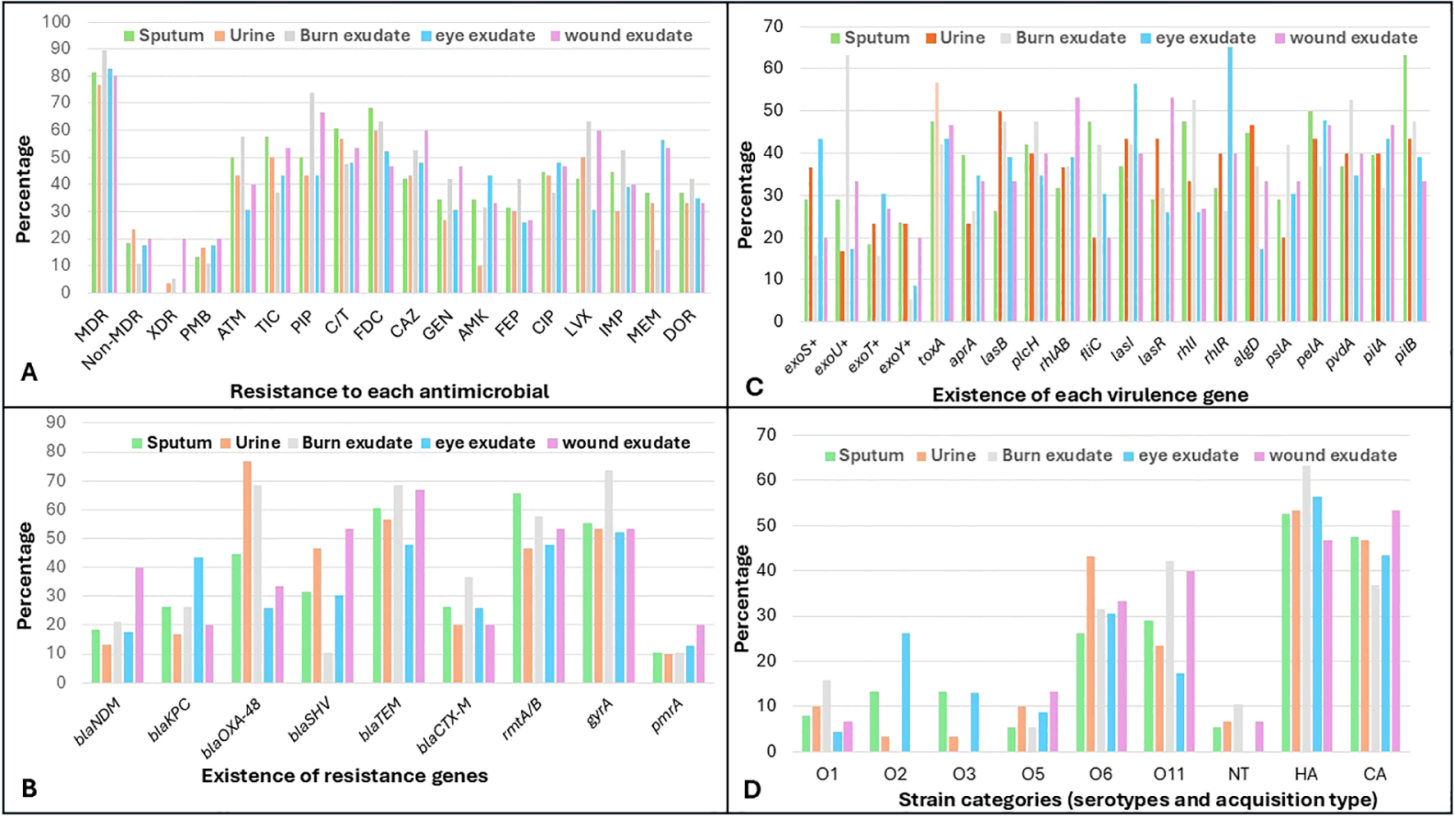
Percentage distribution of resistance profiles, virulence determinants, and serotypes of *Pseudomonas aeruginosa* isolates across different clinical specimen types. **(A)** presents resistance to each antimicrobial and resistance category, including non-MDR (non-multidrug resistant), MDR (multidrug resistant), XDR (extensively drug resistant), ATM (aztreonam), TIC (ticarcillin–clavulanate), PIP (piperacillin), C/T (ceftolozane–tazobactam), DOR (doripenem), CAZ (ceftazidime), FDC (cefiderocol), GEN (gentamicin), AMK (amikacin), FEP (cefepime), CIP (ciprofloxacin), LVX (levofloxacin), IMP (imipenem), and MEM (meropenem). **(B)** shows the prevalence of resistance genes, including *bla*_NDM_ (New Delhi metallo-β-lactamase), *bla*_KPC_ (Klebsiella pneumoniae carbapenemase), *bla*_OXA-48_ (oxacillinase-48), *bla*_SHV_ (sulfhydryl variable β-lactamase), *bla*_TEM_ (Temoneira β-lactamase), *bla*_CTX-M_ (cefotaximase), *rmtA* (16S rRNA methyltransferases A), *gyrA* (DNA gyrase subunit A mutation), and *pmrA* (polymyxin resistance regulator). **(C)** shows virulence gene distribution, including *exoS, exoU, exoT*, and *exoY* (type III secretion system effectors), *toxA* (exotoxin A), *aprA* (alkaline protease), *lasB* (elastase), *plcH* (hemolytic phospholipase C), *rhlAB* (rhamnolipid biosynthesis genes), *fliC* (flagellin), *lasI* and *lasR* (quorum sensing regulators), *rhlI* and *rhlR* (quorum sensing regulators), *algD* (alginate biosynthesis), *pslA* and *pelA* (biofilm formation genes), *pvdA* (pyoverdine synthesis), and *pilA* and *pilB* (type IV pili genes). **(D)** shows serotype distribution based on O-antigen, including O1, O2, O3, O5, O6, O11, NT (non-typeable strains) CA (community acquired isolates), HA (Hospital acquired isolates).

Resistance patterns differed clearly by specimen type. Burn exudate isolates showed high resistance across multiple antibiotic classes, with particularly elevated non-susceptibility to piperacillin at 73.7%, Cefiderocol at 63.2%, levofloxacin at 63.2%, ceftazidime at 52.6%, and imipenem at 52.6%, indicating a substantial multidrug resistance burden. Wound exudate isolates exhibited a similar profile, with high resistance to piperacillin at 66.7%, ceftazidime and levofloxacin each at 60%, Ceftolozane/Tazobactam at 53.3%, and meropenem at 53.3%. Respiratory isolates from sputum samples also demonstrated marked resistance, particularly to third-generation cephalosporins, including Cefiderocol at 68.4% and Ceftolozane/Tazobactam at 60.5%, as well as notable resistance to Ticarcillin/clavulanate at 57.9% and imipenem at 44.7%. In contrast, urinary isolates showed intermediate resistance levels, with resistance to Cefiderocol and Ceftolozane/Tazobactam each at 60%, levofloxacin at 50%, and lower resistance to carbapenems, including imipenem at 30% and meropenem at 33.3%. Eye exudate isolates generally exhibited lower resistance to most agents, although resistance to Cefiderocol remained substantial at 52.2% and meropenem at 56.5%. Across all specimen types, resistance to polymyxin B remained comparatively low, ranging from 10.5% in burn isolates to 20% in wound isolates (**Figure 4A**).

### Resistance genes profiling

Analysis of resistance gene distribution showed high prevalence of β-lactamase–encoding genes, with *bla*_TEM_ detected in 59.2% of isolates and *bla*_OXA-48_ in 51.2%. *bla*_SHV_ was present in 34.4% of isolates, while *bla*_KPC_ and *bla*_NDM_ were detected at lower frequencies of 26.4% and 20%, respectively. The *bla*_CTX-M_ gene occurred in 25.6% of isolates. Non–β-lactam resistance determinants were also common, including mutated *gyrA* in 56.8% of isolates and *rmtA/B* in 55.2%, whereas *pmrA* was detected infrequently at 12%. The frequent coexistence of multiple resistance genes within single isolates provides a genetic basis for the multidrug-resistant phenotypes observed (**Figure 2B**).

Analysis of resistance gene distribution showed widespread detection of β-lactamase genes across all T3SS genotypes. *bla*_TEM_ was the most prevalent determinant, detected in 65.8% of exoS+ and 59.5% of exoU+ isolates, while *bla*_OXA-48_ was also highly prevalent, occurring in over half of exoS+ and exoY+ isolates. In contrast, *bla*_CTX-M_ and *bla*_NDM_ were detected at substantially lower frequencies across genotypes. Mutational resistance mechanisms were prominent, with *gyrA* mutations identified in 62.2% of exoU+ isolates and *rmtA/B* detected in 68.2% of exoY+ isolates. Overall, exoU+ isolates tended to carry multiple resistance determinants simultaneously, whereas exoT+ and exoY+ isolates showed more variable gene combinations rather than uniformly high prevalence (**Figure 3B**).

When stratified by specimen type, *bla*_TEM_ and *bla*_OXA-48_ remained the dominant resistance genes across all sources. Burn exudate isolates showed high carriage of *bla*_TEM_ and *bla*_OXA-48_, each detected in 68.4% of cases, together with frequent mutated *gyrA* at 73.7% and *rmtA/B* at 57.9%. Sputum isolates likewise demonstrated substantial resistance gene burden, with *bla*_TEM_ present in 60.5% and *rmtA/B* and mutated *gyrA* detected in 65.8% and 55.3% of isolates, respectively. Urine isolates exhibited the highest prevalence of *bla*_OXA-48_ at 76.7%, alongside moderate frequencies of *bla*_TEM_ and mutated *gyrA*. Wound exudate isolates showed frequent detection of *bla*_TEM_ at 66.7% and *bla*_SHV_ at 53.3%, accompanied by mutated *gyrA* and *rmtA/B* each at 53.3%. In contrast, eye exudate isolates displayed lower and more heterogeneous gene distributions, although mutated *gyrA* remained common at 52.2%. Across all specimen types, *pmrA* occurred infrequently, remaining at or below 20%. Multiple resistance genes were commonly detected within the isolates of same specimen types, indicating combined resistance mechanisms rather than reliance on a single gene (**Figure 4B**).

### Distribution of virulence determinants

Analysis of virulence gene distribution showed widespread carriage of multiple pathogenic determinants. Among toxin- and protease-related genes, *toxA* and *lasB* were frequently detected, occurring in 48% and 38.4% of isolates, respectively. Quorum-sensing regulators were also common, with *lasI* present in 43.2% of isolates and *rhlR* detected in 40%. Biofilm- and adhesion-associated genes were prominent, particularly *pelA* at 45.6% and *pilB* at 48%, supporting the capacity for surface attachment and persistence. Motility-related genes such as *fliC* were detected in 33.6% of isolates. Taken together, the data revealed that virulence gene distribution was shared among isolates rather than harboring a single factor (**Figure 2C**).

The exoS+ isolates showed higher frequencies of quorum-sensing and regulatory genes, particularly *lasI* at 63.2%, *rhlI* at 50%, and *rhlR* at 52.6%, indicating enhanced regulatory capacity. The exoU+ isolates were enriched for tissue-damaging and cytotoxic determinants, including *toxA* detected in 54.1% and *plcH* in 51.4% of isolates, along with elevated *fliC* carriage at 48.6%. The exoT+ and exoY+ isolates carried core virulence genes at variable levels, including *pilB* detected in 57.1% of exoT+ isolates and *pilA* present in 36.4% of exoY+ isolates, reflecting heterogeneous but conserved virulence potential. The lack of virulence profiles exclusive to a single T3SS genotype does not support strict segregation (**Figure 3C**).

Burn and wound exudate isolates showed higher prevalence of several virulence genes linked to tissue damage and persistence. In burn exudates, *lasB* and *plcH* were each detected in 47.4% of isolates, *toxA* and *pslA* in 42.1%, and *pelA* in 36.8%, indicating substantial cytotoxic and biofilm-forming capacity. Wound exudate isolates likewise demonstrated frequent carriage of *rhlAB* at 53.3%, *lasR* at 53.3%, *pelA* at 46.7%, and *toxA* at 46.7%. Sputum isolates showed notable representation of motility and regulatory genes, with *fliC* detected in 47.4% and *rhlI* in 47.4%, alongside *pelA* in 50% and *pilB* in 63.2% of isolates. Urine isolates carried multiple core virulence genes, including *toxA* in 56.7%, *lasB* in 50%, and *lasR* in 43.3%, although at generally moderate frequencies. Eye exudate isolates also retained key virulence determinants, particularly *lasI* at 56.5% and *rhlR* at 65.2%, but showed lower prevalence of several tissue-damaging genes (**Figure 4C**).

### Serotyping and strain categories

Serotype analysis showed predominance of O6 and O11, which together accounted for the majority of typable isolates, with O6 detected in 32.8% and O11 in 28.8%. Other serotypes, including O1, O2, O3, and O5, were each identified at frequencies below 10%, while non-typable strains were uncommon at 5.6%. Most isolates were classified as hospital-acquired at 54.4%, compared with 45.6% community-acquired isolates (**Figure 2D**).

Strain category analysis showed genotype-associated differences in serotype distribution and acquisition source. The exoU+ isolates were more frequently hospital acquired at 67.6% and were predominantly associated with serotypes O6 and O11, detected in 35.1% and 37.8% of isolates, respectively. In contrast, exoS+ isolates displayed a broader serotype distribution, with O2, O3, and O5 each accounting for approximately one quarter of isolates, and a more balanced hospital- and community-acquired profile. The exoT+ isolates were commonly linked to O6 and O11, together comprising nearly 90% of cases, while exoY+ isolates showed enrichment for O6 at 45.5% and a higher proportion of non-typable strains at 13.6%, alongside a predominance of community-acquired isolates (**Figure 3D**).

With the exception of burn isolates, our results indicate that most isolates, including eye isolates, were nearly equally distributed between hospital-acquired and community-acquired infections. The burn exudate isolates were predominantly hospital acquired at 63.2% and were mainly associated with serotypes O11 at 42.1% and O6 at 31.6%. Wound exudate isolates showed a higher proportion of community-acquired strains at 53.3% but remained enriched for O11 at 40% and O6 at 33.3%. Sputum isolates exhibited a near-balanced hospital- and community-acquired distribution, with O11 and O6 detected in 28.9% and 26.3% of isolates, respectively. Urine isolates showed a more even acquisition profile and a distinct enrichment of O6 at 43.3%, while eye exudate isolates displayed greater serotype diversity, including O2 at 26.1% and O3 at 13%, alongside lower representation of O11 (**Figure 4D**).

### Intra-group diversity and clonality of *P. aeruginosa*

#### Cluster analysis of *P. aeruginosa* by T3SS effector genotypes

**Figure 5:**
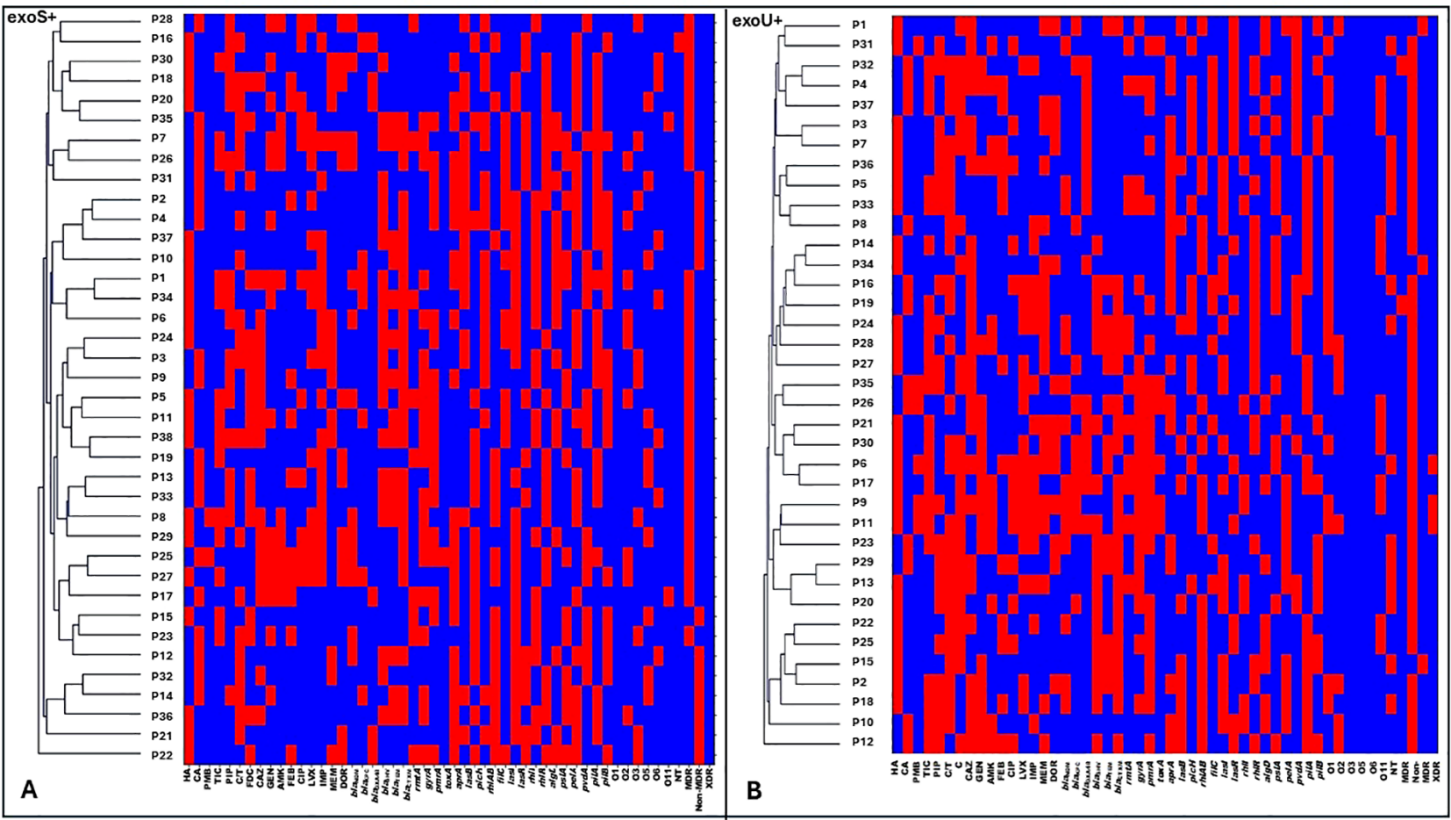
Hierarchical clustering analysis of exoS+ and exoU+ producing *Pseudomonas aeruginosa* isolates based on antimicrobial resistance, resistance gene carriage, virulence gene profiles, and serotype distribution. **(A)** shows hierarchical clustering of exoS+ isolates. **(B)** shows hierarchical clustering of exoU+ isolates. Rows represent individual isolates, and columns represent phenotypic and genotypic variables, Antimicrobial resistance variables, including non-MDR (non-multidrug resistant), MDR (multidrug resistant), XDR (extensively drug resistant), ATM (aztreonam), TIC (ticarcillin–clavulanate), PIP (piperacillin), C/T (ceftolozane–tazobactam), DOR (doripenem), CAZ (ceftazidime), FDC (cefiderocol), GEN (gentamicin), AMK (amikacin), FEP (cefepime), CIP (ciprofloxacin), LVX (levofloxacin), IMP (imipenem), and MEM (meropenem).Resistance genes, including *bla*_NDM_ (New Delhi metallo-β-lactamase), *bla*_KPC_ (Klebsiella pneumoniae carbapenemase), *bla*_OXA-48_ (oxacillinase-48), *bla*_SHV_ (sulfhydryl variable β-lactamase), *bla*_TEM_ (Temoneira β-lactamase), *bla*_CTX-M_ (cefotaximase), *rmtA* (16S rRNA methyltransferases A), *gyrA* (DNA gyrase subunit A mutation), and *pmrA* (polymyxin resistance regulator). Virulence genes, including *exoS, exoU, exoT*, and *exoY* (type III secretion system effectors), *toxA* (exotoxin A), *aprA* (alkaline protease), *lasB* (elastase), *plcH* (hemolytic phospholipase C), *rhlAB* (rhamnolipid biosynthesis genes), *fliC* (flagellin), *lasI* and *lasR* (quorum sensing regulators), *rhlI* and *rhlR* (quorum sensing regulators), *algD* (alginate biosynthesis), *pslA* and *pelA* (biofilm formation genes), *pvdA* (pyoverdine synthesis), and *pilA* and *pilB* (type IV pili genes). Serotype distribution based on O-antigen, including O1, O2, O3, O5, O6, O11, NT (non-typeable strains) CA (community acquired isolates), HA (Hospital acquired isolates). Presence of a trait is shown in red and absence in blue.

**Figure 6:**
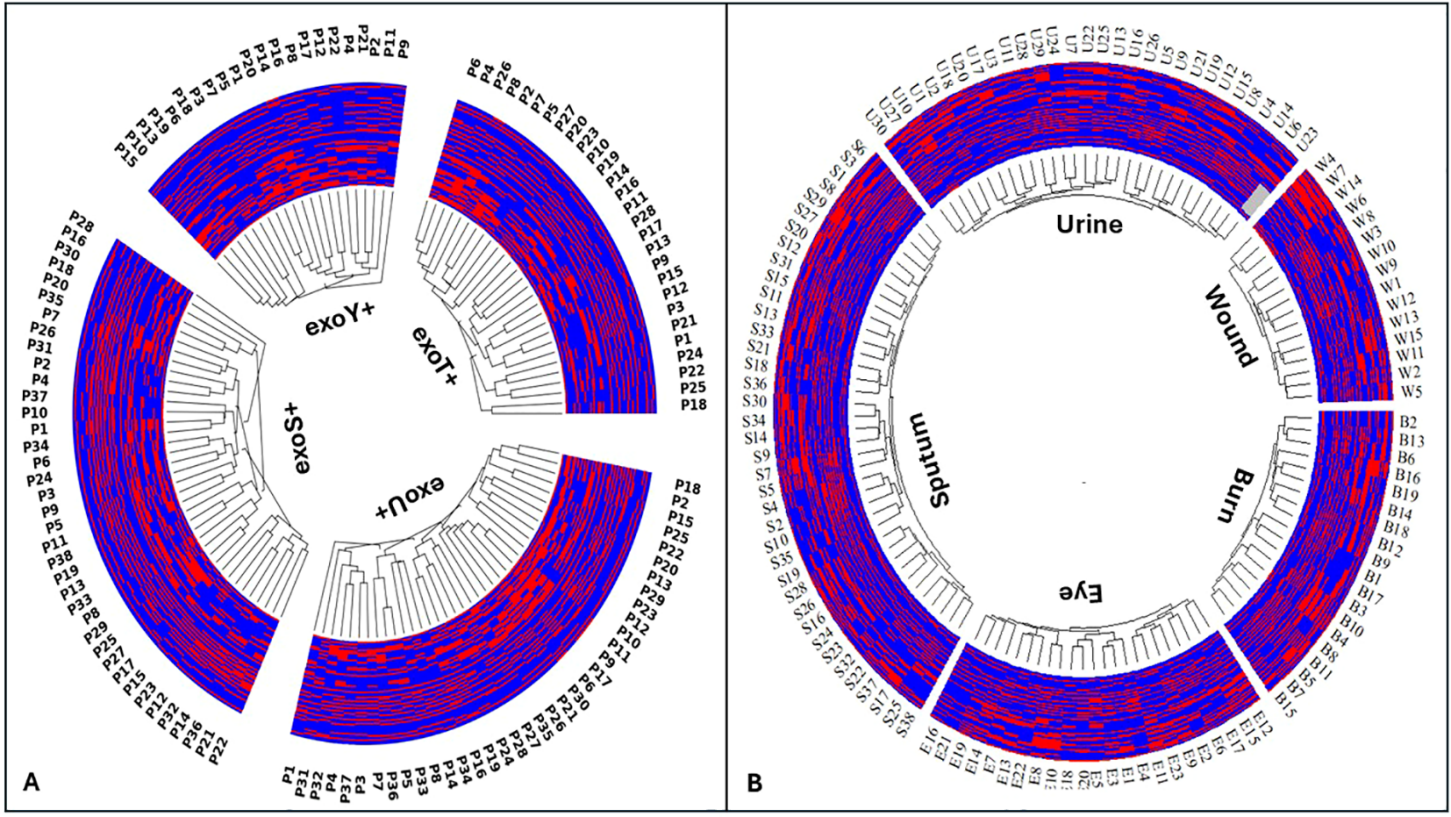
Integrative circular hierarchical clustering of *Pseudomonas aeruginosa i*solates based on combined antimicrobial resistance profiles, resistance gene carriage, virulence gene repertoires, serotypes, and clinical manifestations. **(A)** shows clustering according to type III secretion system effector genotypes (Intra-group typing), including exoS+, exoU+, exoT+, and exoY+. **(B)** shows clustering according to clinical sample types (Intra-group typing), including urine, sputum, wound exudate, burn exudate, and eye exudate. Each branch represents an individual isolate, and concentric heatmap rings represent binary phenotypic and genotypic variables. Presence of a feature is shown in red and absence in blue.

**Figure 7:**
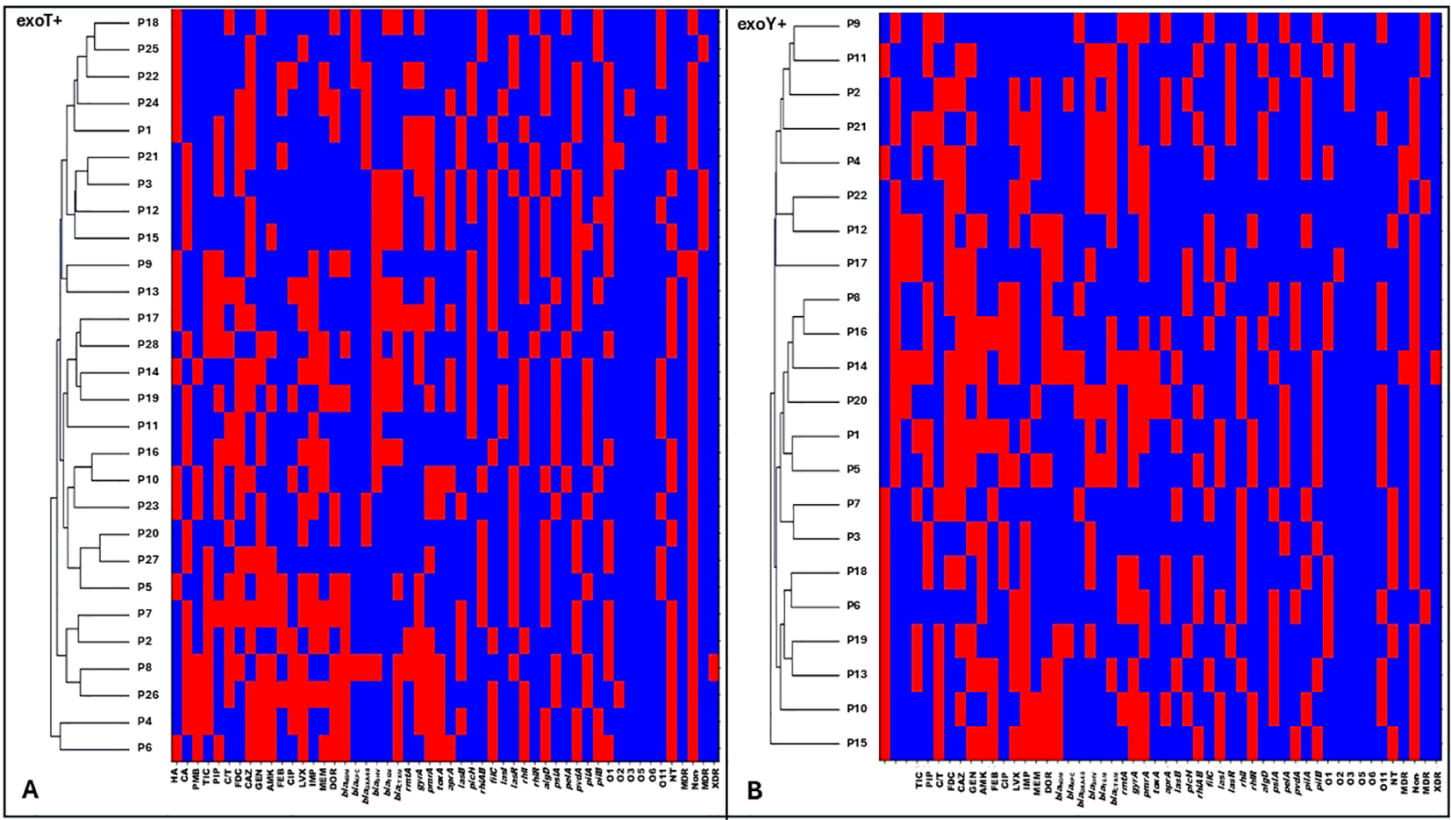
Clustering patterns of antimicrobial resistance, genetic determinants, and serotypes among exoT+ and exoY+ *Pseudomonas aeruginosa* isolates. **(A)** shows hierarchical clustering of exoT+ isolates. **(B)** shows hierarchical clustering of exoY+ isolates. Rows represent individual isolates, and columns represent phenotypic and genotypic variables, Antimicrobial resistance variables, including non-MDR (non-multidrug resistant), MDR (multidrug resistant), XDR (extensively drug resistant), ATM (aztreonam), TIC (ticarcillin–clavulanate), PIP (piperacillin), C/T (ceftolozane–tazobactam), DOR (doripenem), CAZ (ceftazidime), FDC (cefiderocol), GEN (gentamicin), AMK (amikacin), FEP (cefepime), CIP (ciprofloxacin), LVX (levofloxacin), IMP (imipenem), and MEM (meropenem).Resistance genes, including *bla*_NDM_ (New Delhi metallo-β-lactamase), *bla*_KPC_ (Klebsiella pneumoniae carbapenemase), *bla*_OXA-48_ (oxacillinase-48), *bla*_SHV_ (sulfhydryl variable β-lactamase), *bla*_TEM_ (Temoneira β-lactamase), *bla*_CTX-M_ (cefotaximase), *rmtA* (16S rRNA methyltransferases A), *gyrA* (DNA gyrase subunit A mutation), and *pmrA* (polymyxin resistance regulator). Virulence genes, including *exoS, exoU, exoT*, and *exoY* (type III secretion system effectors), *toxA* (exotoxin A), *aprA* (alkaline protease), *lasB* (elastase), *plcH* (hemolytic phospholipase C), *rhlAB* (rhamnolipid biosynthesis genes), *fliC* (flagellin), *lasI* and *lasR* (quorum sensing regulators), *rhlI* and *rhlR* (quorum sensing regulators), *algD* (alginate biosynthesis), *pslA* and *pelA* (biofilm formation genes), *pvdA* (pyoverdine synthesis), and *pilA* and *pilB* (type IV pili genes). Serotype distribution based on O-antigen, including O1, O2, O3, O5, O6, O11, NT (non-typeable strains) CA (community acquired isolates), HA (Hospital acquired isolates). Presence of a trait is shown in red and absence in blue.

Hierarchical clustering based on combined antimicrobial resistance patterns, virulence gene profiles, and serotype distribution revealed pronounced intra-type heterogeneity among *P. aeruginosa* isolates. Within the exoS+ genotype, each isolate formed a separate cluster, reflecting distinct resistance profiles, virulence gene combinations, and serotypes, with no evidence of shared clustering (**Figure 5A**; **Figure 6A**). A similar pattern was observed for exoT+ isolates, where isolates were dispersed across individual clusters, indicating high variability and absence of clonal relatedness (**Figure 7A**; **Figure 6A**). The exoU+ isolates also clustered independently, despite a higher overall resistance burden, confirming that increased resistance did not translate into shared resistance–virulence signatures (**Figure 5B**; **Figure 6A**). The exoY+ isolates showed the same isolate-specific clustering, with predominantly susceptible antimicrobial profiles but distinct virulence and serotype patterns that prevented cluster overlap (**Figure 7B**; **Figure 6A**). Across all types, no cluster harbored more than one isolate, demonstrating that clustering was driven by isolate-specific combinations of antimicrobial resistance, virulence genes, and serotypes rather than type identity.

#### Cluster analysis of *P. aeruginosa* by sample type

**Figure 8:**
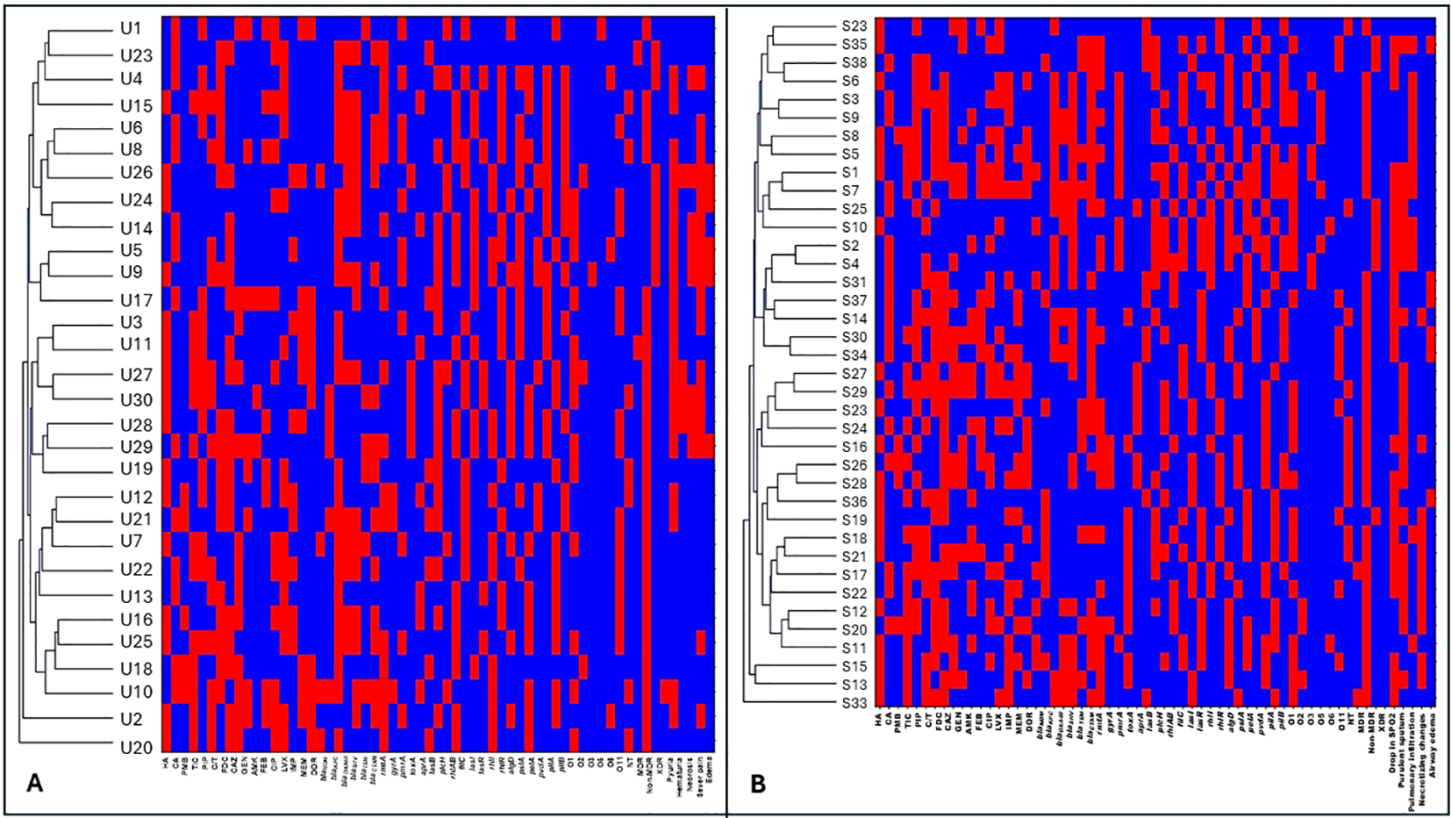
Hierarchical clustering of *Pseudomonas aeruginosa* isolates recovered from urine and sputum specimens. **(A)** shows hierarchical clustering of urine-derived isolates U. **(B)** shows hierarchical clustering of sputum-derived isolates S. Rows represent individual isolates, and columns represent phenotypic and genotypic variables, Antimicrobial resistance variables, including non-MDR (non-multidrug resistant), MDR (multidrug resistant), XDR (extensively drug resistant), ATM (aztreonam), TIC (ticarcillin–clavulanate), PIP (piperacillin), C/T (ceftolozane–tazobactam), DOR (doripenem), CAZ (ceftazidime), FDC (cefiderocol), GEN (gentamicin), AMK (amikacin), FEP (cefepime), CIP (ciprofloxacin), LVX (levofloxacin), IMP (imipenem), and MEM (meropenem).Resistance genes, including *bla*_NDM_ (New Delhi metallo-β-lactamase), *bla*_KPC_ (Klebsiella pneumoniae carbapenemase), *bla*_OXA-48_ (oxacillinase-48), *bla*_SHV_ (sulfhydryl variable β-lactamase), *bla*_TEM_ (Temoneira β-lactamase), *bla*_CTX-M_ (cefotaximase), *rmtA* (16S rRNA methyltransferases A), *gyrA* (DNA gyrase subunit A mutation), and *pmrA* (polymyxin resistance regulator). Virulence genes, including *exoS, exoU, exoT*, and *exoY* (type III secretion system effectors), *toxA* (exotoxin A), *aprA* (alkaline protease), *lasB* (elastase), *plcH* (hemolytic phospholipase C), *rhlAB* (rhamnolipid biosynthesis genes), *fliC* (flagellin), *lasI* and *lasR* (quorum sensing regulators), *rhlI* and *rhlR* (quorum sensing regulators), *algD* (alginate biosynthesis), *pslA* and *pelA* (biofilm formation genes), *pvdA* (pyoverdine synthesis), and *pilA* and *pilB* (type IV pili genes). Serotype distribution based on O-antigen, including O1, O2, O3, O5, O6, O11, NT (non-typeable strains) CA (community acquired isolates), HA (Hospital acquired isolates). Presence of a trait is shown in red and absence in blue.

Hierarchical clustering integrating antimicrobial resistance, virulence gene profiles, serotypes, and clinical metadata demonstrated strong heterogeneity across all sample types. Urine isolates clustered individually, displaying heterogeneous antimicrobial resistance profiles and variable virulence gene content. Although some urine isolates showed increased resistance to selected antibiotics, these traits did not translate into shared clustering, indicating absence of niche-specific clonal expansion (**Figure 8A**; **Figure 6B**). Sputum isolates exhibited a comparable pattern, with isolates distributed across separate clusters and no dominant resistance–virulence signature associated with the respiratory niche (**Figure 8B**; **Figure 6B**).

**Figure 9:**
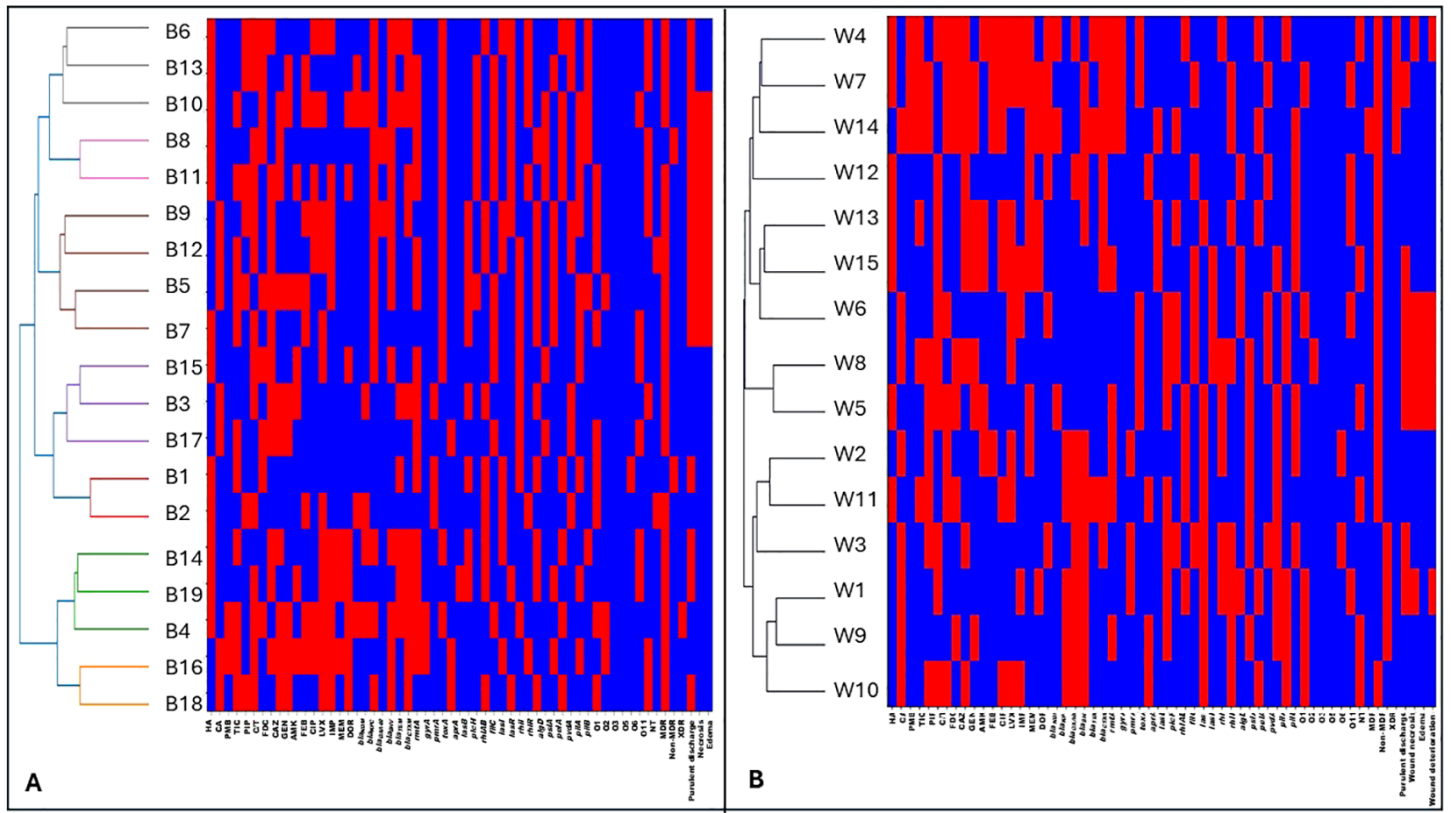
Hierarchical clustering of burn- and wound-derived *Pseudomonas aeruginosa* isolates based on integrated phenotypic and genotypic profiles. **(A)** shows clustering of burn-derived isolates **(B)** B. shows clustering of wound-derived isolates W. Rows represent individual isolates, and columns represent binary phenotypic and genotypic variables. Antimicrobial resistance variables, including non-MDR (non-multidrug resistant), MDR (multidrug resistant), XDR (extensively drug resistant), ATM (aztreonam), TIC (ticarcillin–clavulanate), PIP (piperacillin), C/T (ceftolozane–tazobactam), DOR (doripenem), CAZ (ceftazidime), FDC (cefiderocol), GEN (gentamicin), AMK (amikacin), FEP (cefepime), CIP (ciprofloxacin), LVX (levofloxacin), IMP (imipenem), and MEM (meropenem).Resistance genes, including *bla*_NDM_ (New Delhi metallo-β-lactamase), *bla*_KPC_ (Klebsiella pneumoniae carbapenemase), *bla*_OXA-48_ (oxacillinase-48), *bla*_SHV_ (sulfhydryl variable β-lactamase), *bla*_TEM_ (Temoneira β-lactamase), *bla*_CTX-M_ (cefotaximase), *rmtA* (16S rRNA methyltransferases A), *gyrA* (DNA gyrase subunit A mutation), and *pmrA* (polymyxin resistance regulator). Virulence genes, including *exoS, exoU, exoT*, and *exoY* (type III secretion system effectors), *toxA* (exotoxin A), *aprA* (alkaline protease), *lasB* (elastase), *plcH* (hemolytic phospholipase C), *rhlAB* (rhamnolipid biosynthesis genes), *fliC* (flagellin), *lasI* and *lasR* (quorum sensing regulators), *rhlI* and *rhlR* (quorum sensing regulators), *algD* (alginate biosynthesis), *pslA* and *pelA* (biofilm formation genes), *pvdA* (pyoverdine synthesis), and *pilA* and *pilB* (type IV pili genes). Serotype distribution based on O-antigen, including O1, O2, O3, O5, O6, O11, NT (non-typeable strains) CA (community acquired isolates), HA (Hospital acquired isolates). Presence of a trait is shown in red and absence in blue.

**Figure 10:**
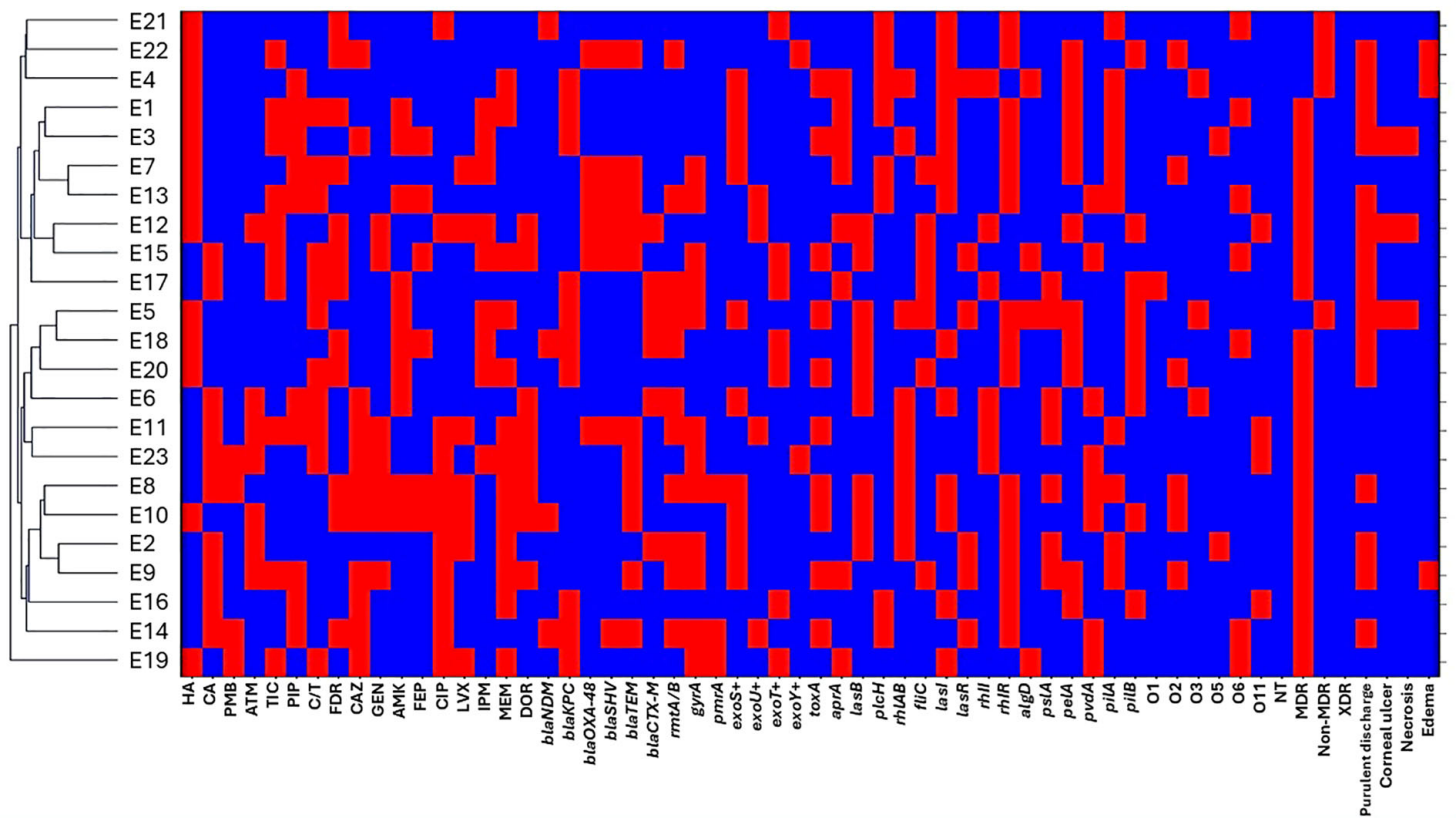
Hierarchical clustering of *Pseudomonas aeruginosa* isolates recovered from eye exudates. Rows represent individual eye-derived isolates E, and columns represent binary phenotypic and genotypic variables. Antimicrobial resistance variables, including non-MDR (non-multidrug resistant), MDR (multidrug resistant), XDR (extensively drug resistant), ATM (aztreonam), TIC (ticarcillin–clavulanate), PIP (piperacillin), C/T (ceftolozane–tazobactam), DOR (doripenem), CAZ (ceftazidime), FDC (cefiderocol), GEN (gentamicin), AMK (amikacin), FEP (cefepime), CIP (ciprofloxacin), LVX (levofloxacin), IMP (imipenem), and MEM (meropenem).Resistance genes, including *bla*_NDM_ (New Delhi metallo-β-lactamase), *bla*_KPC_ (Klebsiella pneumoniae carbapenemase), *bla*_OXA-48_ (oxacillinase-48), *bla*_SHV_ (sulfhydryl variable β-lactamase), *bla*_TEM_ (Temoneira β-lactamase), *bla*_CTX-M_ (cefotaximase), *rmtA* (16S rRNA methyltransferases A), *gyrA* (DNA gyrase subunit A mutation), and *pmrA* (polymyxin resistance regulator). Virulence genes, including *exoS, exoU, exoT*, and *exoY* (type III secretion system effectors), *toxA* (exotoxin A), *aprA* (alkaline protease), *lasB* (elastase), *plcH* (hemolytic phospholipase C), *rhlAB* (rhamnolipid biosynthesis genes), *fliC* (flagellin), *lasI* and *lasR* (quorum sensing regulators), *rhlI* and *rhlR* (quorum sensing regulators), *algD* (alginate biosynthesis), *pslA* and *pelA* (biofilm formation genes), *pvdA* (pyoverdine synthesis), and *pilA* and *pilB* (type IV pili genes). Serotype distribution based on O-antigen, including O1, O2, O3, O5, O6, O11, NT (non-typeable strains) CA (community acquired isolates), HA (Hospital acquired isolates). Presence of a trait is shown in red and absence in blue.

Blood isolates showed wide dispersion across the dendrogram, with each isolate forming an independent cluster, indicating high diversity in resistance and virulence profiles and no evidence of clonality (**Figure 9A**; **Figure 6B**). Resistance patterns varied across multiple antibiotic classes, and virulence gene carriage differed markedly between isolates, suggesting independent acquisition events rather than bloodstream adaptation driving similarity. Wound isolates followed the same trend, with marked variability in resistance, virulence genes, and serotypes and no cluster harboring more than one isolate (**Figure 9B**; **Figure 6B**). For eye isolates, resistant and susceptible profiles were interspersed throughout the dendrogram, with no dominant resistance pattern defining any cluster (**Figure 10**; **Figure 6B**). Isolates with similar resistance to individual antibiotics did not consistently cluster together, indicating that resistance traits were distributed independently across the population. Moreover, no conserved resistance or virulence gene set characterized any cluster.

Isolates from invasive sources such as blood and respiratory samples tended to display a higher number of antimicrobial resistance traits and a broader repertoire of virulence genes compared with isolates from urine or wound samples. Urine and wound isolates generally showed fewer antimicrobial resistance markers and a more limited virulence gene content, although exceptions were present. Certain serotypes appeared more frequently among respiratory and blood isolates, but their distribution was scattered and not restricted to specific clusters or resistance profiles. Importantly, no sample type exhibited exclusive resistance patterns, virulence gene combinations, or serotype signatures. Each isolate retained a distinct profile, indicating that sample type influenced trait frequency but did not define isolate identity.

### Inter-group phylogenetic analysis of *P. aeruginosa*

**Figure 11:**
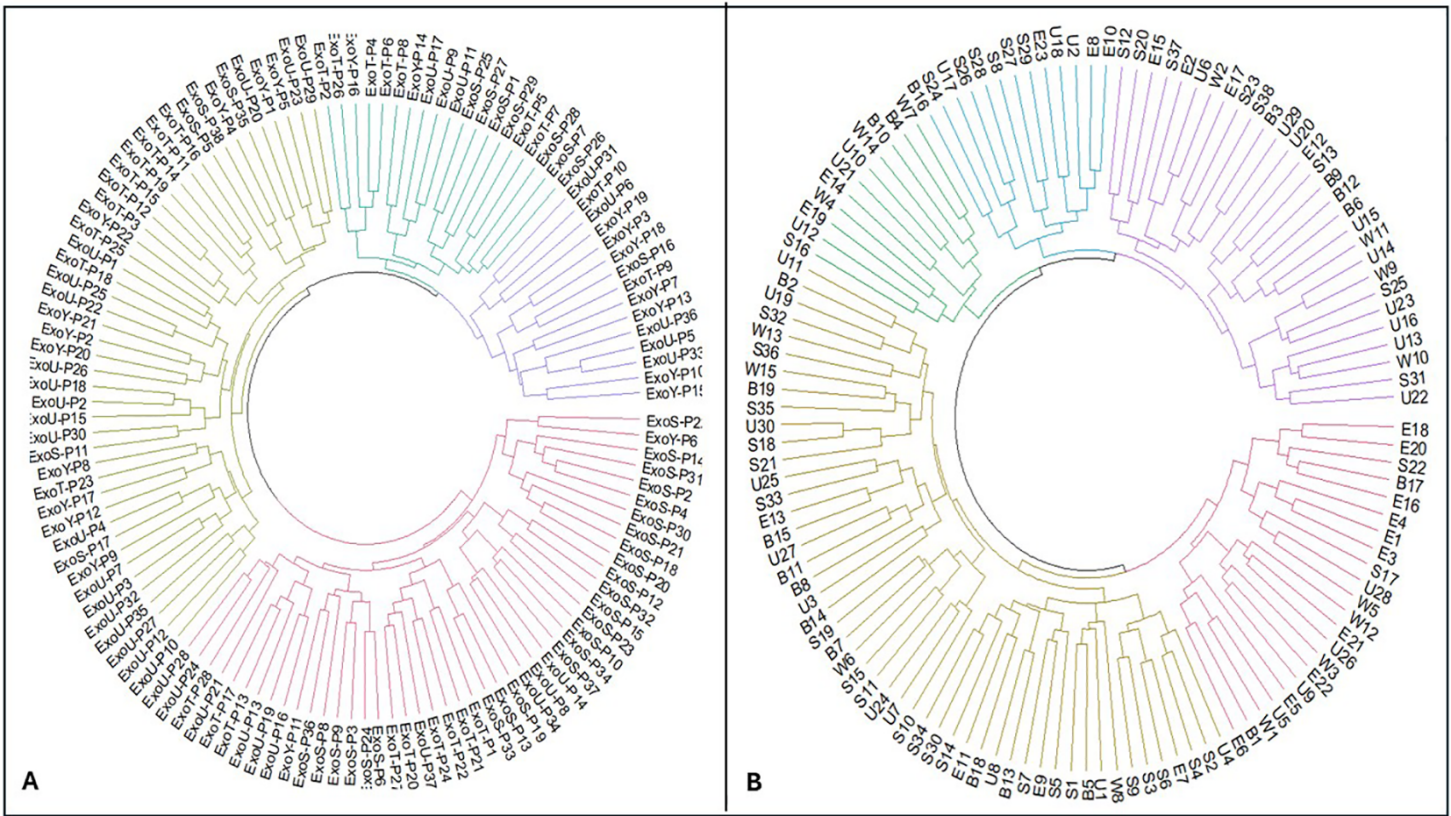
Circular inter-group hierarchical clustering of *Pseudomonas aeruginosa* isolates based on overall genetic relatedness. **(A)** shows inter-group typing according to type III secretion system effector genotypes, including exoS+, exoU+, exoT+, and exoY+. **(B)** shows inter-group typing according to clinical sample origin, including urine U, sputum S, wound exudate W, burn exudate B, and eye exudate E. Each terminal node represents a single isolate, and branch lengths reflect relative genetic similarity derived from integrated phenotypic and genotypic data. Clustering is informed by combined antimicrobial resistance patterns, resistance gene profiles, virulence gene repertoires, serotypes, and clinical manifestations. Colored branches indicate major strain groupings and highlight intra- and inter-group relatedness among isolates across T3SS effector backgrounds and sample types.

The circular dendrogram (**Figure 11A**) showed that *P. aeruginosa* isolates were grouped according to their T3SS effector genotypes. The exoT+ and exoY+ genotypes were widely distributed and frequently overlapped across the same branches, reflecting their common and conserved presence among isolates. In contrast, overlap between exoS+ and exoU+ genotypes was minimal. These two genotypes were largely separated into distinct clusters, with only rare instances of close branching. Most isolates carrying the exoU+ genotype grouped within the same lineage, while isolates carrying the exoS+ genotype formed a separate lineage, showing limited overlap between these two genotypes.

The phylogenetic tree (**Figure 11B**) illustrated the distribution of *P. aeruginosa* isolates based on their isolation sites. These isolates from different clinical sources showed partial overlap, with some source specific clustering and some mixed grouping across the dendrogram. Burn isolates (labeled B) clustered together in several closely related branches, indicating genetic similarity among these isolates. Wound isolates (labeled W) were distributed across multiple branches and showed partial overlap with burn isolates, forming mixed clusters. Sputum isolates (labeled S) formed distinct clusters but were also interspersed with other sources, showing moderate diversity. Urine isolates (labeled U) grouped into several related branches and overlapped with wounds and sputum isolates in some clusters. Eye exudate isolates (labeled E) were mainly grouped within specific branches but also appeared alongside other isolate types.

### Correlation analysis of resistance, virulence, and clinical features across *P. aeruginosa* infection types

**Figure 12:**
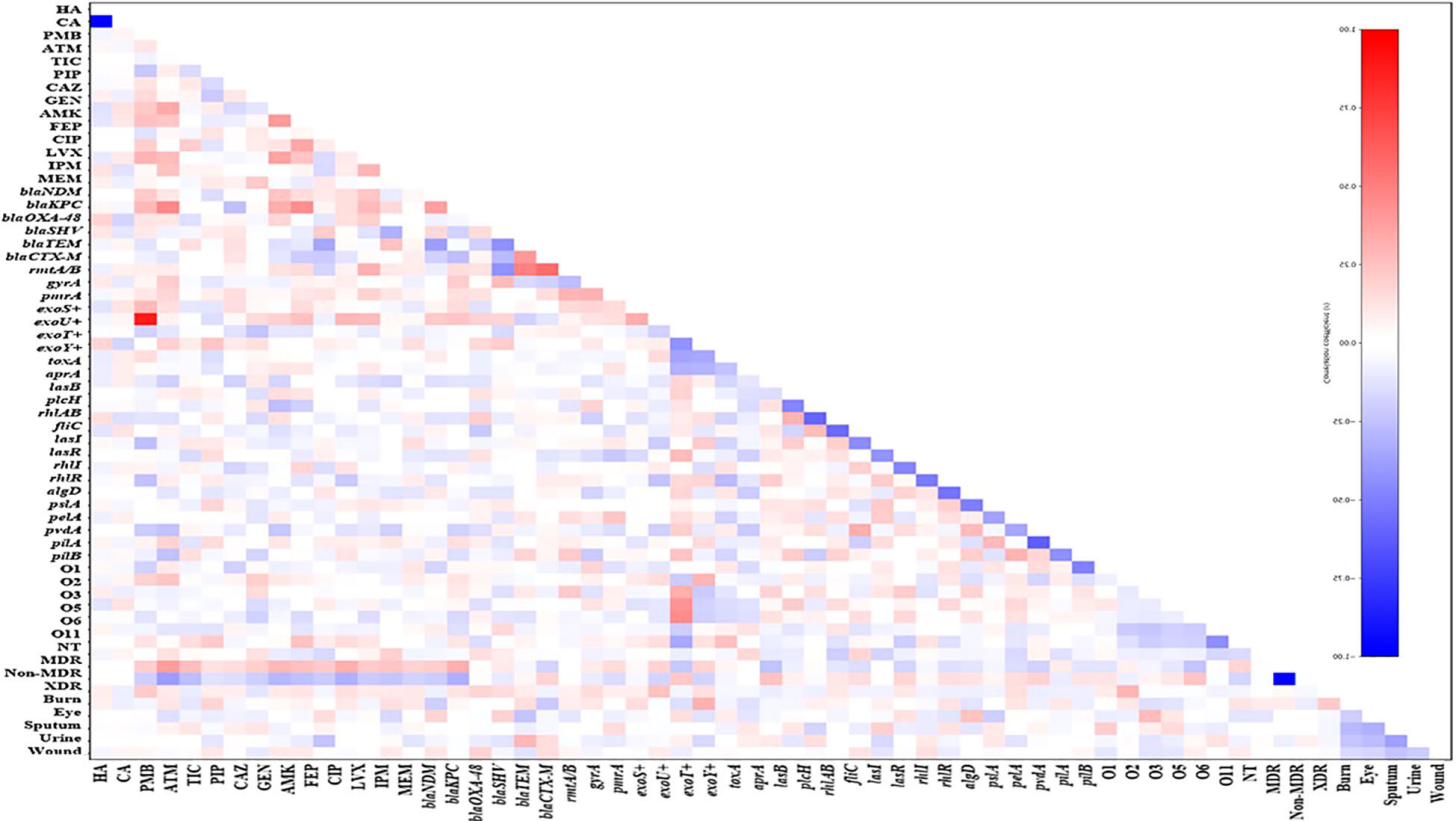
Correlation analysis of antimicrobial resistance phenotypes, resistance genes, virulence determinants, serotypes, resistance categories, and clinical sample types among *Pseudomonas aeruginosa* isolates. The triangular heatmap displays pairwise correlation coefficients calculated across all studied variables. Variables include resistance to antimicrobials, including non-MDR (non-multidrug resistant), MDR (multidrug resistant), XDR (extensively drug resistant), ATM (aztreonam), TIC (ticarcillin–clavulanate), PIP (piperacillin), C/T (ceftolozane–tazobactam), DOR (doripenem), CAZ (ceftazidime), FDC (cefiderocol), GEN (gentamicin), AMK (amikacin), FEP (cefepime), CIP (ciprofloxacin), LVX (levofloxacin), IMP (imipenem), and MEM (meropenem).Resistance genes, including *bla*_NDM_ (New Delhi metallo-β-lactamase), *bla*_KPC_ (Klebsiella pneumoniae carbapenemase), *bla*_OXA-48_ (oxacillinase-48), *bla*_SHV_ (sulfhydryl variable β-lactamase), *bla*_TEM_ (Temoneira β-lactamase), *bla*_CTX-M_ (cefotaximase), *rmtA* (16S rRNA methyltransferases A), *gyrA* (DNA gyrase subunit A mutation), and *pmrA* (polymyxin resistance regulator). Virulence genes, including *exoS, exoU, exoT*, and *exoY* (type III secretion system effectors), *toxA* (exotoxin A), *aprA* (alkaline protease), *lasB* (elastase), *plcH* (hemolytic phospholipase C), *rhlAB* (rhamnolipid biosynthesis genes), *fliC* (flagellin), *lasI* and *lasR* (quorum sensing regulators), *rhlI* and *rhlR* (quorum sensing regulators), *algD* (alginate biosynthesis), *pslA* and *pelA* (biofilm formation genes), *pvdA* (pyoverdine synthesis), and *pilA* and *pilB* (type IV pili genes). Serotype distribution based on O-antigen, including O1, O2, O3, O5, O6, O11, NT (non-typeable strains) CA (community acquired isolates), HA (Hospital acquired isolates). Clinical sample sources burn, eye, sputum, urine, and wound. Color intensity represents the strength and direction of correlation, with red indicating positive correlations and blue indicating negative correlations, according to the scale bar showing correlation coefficient values.

Pearson correlation analysis showed a scattered pattern of positive and negative associations across antimicrobial resistance, resistance genes, virulence factors, serotypes, and epidemiological variables. Positive correlations were mainly observed among related resistance phenotypes, such as resistance to ciprofloxacin correlating with resistance to levofloxacin and carbapenems, and between MDR and XDR classifications, reflecting accumulation of resistance traits. Negative correlations largely involved mutually exclusive categories, including the strong inverse relationship between non-MDR and MDR/XDR status and between hospital- and community-acquired classifications (**Figure 12**). Resistance genes showed weak and inconsistent correlations with phenotypic resistance; for example, beta-lactamase genes *bla*_CTX-M_*and bla*_OXA_ variants showed modest positive associations with beta-lactam resistance, but no resistance gene was strongly or exclusively linked to a specific resistance phenotype. T3SS effector genes showed mixed correlations with resistance and virulence traits, with exoU+ displaying weak positive correlations with MDR and some resistance phenotypes, while exoS+, exoT+, and exoY+ showed generally low and inconsistent correlations, indicating that effector distribution is largely independent of resistance burden. Virulence genes also showed weak and variable correlations with resistance and epidemiological variables; quorum-sensing genes *lasI, lasR, rhlI, and rhlR* and adhesion and biofilm genes *pilA, pilB, algD, pslA, and pelA* were distributed across eye, blood, urine, and wound isolates without forming source- or resistance-specific clusters. Serotypes O1, O2, O3, O5, O6, and O11 showed mixed correlations with resistance and virulence traits, with no consistent association with MDR/XDR status, T3SS effector profiles, or infection origin. Similarly, sample origin displayed heterogeneous patterns, with blood and respiratory isolates showing modest enrichment of MDR/XDR isolates, while eye, urine, and wound isolates remained highly diverse, supporting independent distribution of resistance, virulence, serotype, and epidemiological features (**Figure 12**).

Correlation analysis across burn, eye, sputum, urine, and wound isolates identified a consistent pattern linking exoU+ with markers of clinical severity. Across all specimen types, exoU+ showed positive correlations with tissue-damaging and inflammatory manifestations, including purulent discharge, necrosis, edema, corneal ulceration, pulmonary infiltration, airway edema, pyuria, hematuria, and wound deterioration. These associations indicate that exoU+ isolates are more frequently linked to severe local tissue injury rather than mild disease. In contrast, exoS+ showed mixed and inconsistent correlations with clinical findings across all sample types, occurring in both severe and less severe infections without a stable clinical signature. The correlation analysis revealed variable associations between the exoT+ and exoY+ genotypes and clinical manifestations across the different infection sources (**Supplementary Figures 1**, **2**). In several isolate groups, particularly those obtained from sputum, burn, and wound samples, exoT showed moderate positive correlations with severe clinical manifestations, including necrosis, tissue inflammation, and purulent discharge. In contrast, the exoY genotype demonstrated weaker and more variable correlations with clinical manifestations, although mild positive associations were observed with inflammatory symptoms such as edema and purulent exudate in certain infection sites. Across specimen types, MDR and XDR classifications showed modest positive correlations with severe clinical features, indicating enrichment of resistant isolates in more severe infections, but no single resistance phenotype or gene dominated any clinical presentation. Virulence genes related to quorum sensing and biofilm formation were broadly distributed and did not define specimen-specific clinical patterns (**Supplementary Figures 1**, **2**).

## Discussion

The spread of infectious diseases is a major public health problem. It causes more people to get sick and more people to die (Elmanakhly et al., 2022; Bendary et al., 2022). It puts pressure on hospitals and clinics, leading to crowded wards and limited staff and supplies. It can delay regular care and increase costs. It can also spread inside hospitals and increase antibiotic use, which can lead to antibiotic resistance and harder treatment (Abousaty et al., 2024; Ghaly et al., 2023). The recovery rate of *P. aeruginosa* in this study (25%) falls within the upper range reported by hospital-based studies conducted in high-risk settings, where prevalence values of approximately 17–30% have been documented, particularly in tertiary care hospitals and ICU-enriched patient populations (Maharjan, 2022). High prevalence of *P. aeruginosa* could be attributed to poor hygiene, use of mechanical ventilation, and frequent use of invasive devices such as endotracheal tubes and catheters, which encourage biofilm formation and persistence on surfaces and equipment (Taner et al., 2024). Moreover, hospital water systems, including taps and drains, and shared medical equipment act as environmental reservoirs for *P. aeruginosa*, facilitating survival and transmission to patients. The predominance of the exoS+ genotype at 30.4% is consistent with multiple studies showing exoS+ as the dominant type III secretion effector in invasive and persistent infections, especially in urinary and ocular sources where long-term colonization and epithelial invasion are favored (Horna et al., 2019). The comparable distribution of exoS+ and exoU+ in sputum supports previous observations that respiratory infections can serve as a shared niche for both invasive and cytotoxic phenotypes (Qin et al., 2022). In contrast, the strong association of exoU+ with burn exudates aligns with well documented evidence that exoU+ strains dominate in acute tissue damage environments where rapid cytotoxicity provides a selective advantage, leading to severe inflammation and poor clinical outcomes (Hardy et al., 2022). This pattern suggests that virulence types are selected by specific infection sites rather than spreading at random, with exoS+ linked to persistence and spread, and exoU+ favored in severe tissue damage such as burn wounds.

Most isolates were classified as hospital-acquired (54.4%) compared with community-acquired isolates (45.6%), which is consistent with the recognized role of *P. aeruginosa* as a major nosocomial pathogen in environments with high antibiotic pressure, invasive procedures, and prolonged hospitalization (Hauser, 2009; Centers for Disease Control and Prevention, 2023). The higher proportion of exoU+ isolates among hospital-acquired cases aligns with previous reports showing that exoU+ is strongly associated with ICU settings and severe healthcare-associated infections, where its phospholipase activity drives rapid cell death, tissue necrosis, and poor clinical outcomes (Foulkes et al., 2019; Nolasco-Romero et al., 2024). In contrast, the more balanced distribution of exoS+ among hospital- and community-acquired isolates reflects its association with persistence and invasive but less destructive infections, allowing exoS+ strains to circulate across both community and healthcare settings (Hauser, 2009). These findings indicated that hospital environments preferentially select highly cytotoxic exoU+ strains, whereas exoS+ strains exhibit broader ecological adaptability.

With the exception of burn isolates, most isolates in this study, including those obtained from eye infections, were nearly equally distributed between hospital-acquired and community-acquired infections. Although ocular infections are generally considered more likely to be community-acquired, the relatively high proportion of hospital-acquired eye isolates observed in this study may be explained by the clinical characteristics of the patient population and exposure to healthcare-related procedures. Hospitalized patients frequently undergo ophthalmic examinations, surgical procedures, and topical treatments that may increase the risk of acquiring opportunistic pathogens such as *P. aeruginosa* (Javanmardi et al., 2019). In addition, prolonged hospitalization and underlying diseases may facilitate the transmission of microorganisms within healthcare environments. Previous studies have shown that *P. aeruginosa* can persist in moist hospital reservoirs, including medical equipment, sinks, and ophthalmic solutions, contributing to healthcare-associated infections (Hauser, 2009; Church et al., 2006). Furthermore, ocular infections associated with medical interventions or contaminated ophthalmic materials have been reported in hospital settings, which may increase the proportion of hospital-acquired eye isolates in certain clinical studies (Stapleton and Carnt, 2012). These factors may explain the relatively higher proportion of hospital-acquired ocular isolates observed in the present study. In contrast, burn infections are frequently associated with hospital environments due to prolonged hospitalization, extensive tissue damage, and exposure to contaminated hospital equipment or surfaces, which facilitate colonization by opportunistic pathogens such as *P. aeruginosa*.

Our results showed that the exoU+ genotype was consistently associated with severe pathological features across all anatomical sites, with extensive tissue and wound destruction observed almost exclusively in exoU+ infections. This genotype shows a strong association with tissue necrosis, rapid cell death, and severe clinical outcomes regardless of specimen type, reflecting the potent phospholipase activity of the EXOU effector protein that directly disrupts host cell membranes. In contrast, non-exoU+ isolates, most commonly exoS+ strains, were mainly associated with localized inflammation and less destructive invasive pathology. Experimental and clinical evidence indicates that EXOU drives acute cytotoxic injury independent of infection site, whereas EXOS protein promotes persistence and spread with comparatively limited tissue damage, supporting the conclusion that effector genotype is a stronger determinant of disease severity than anatomical location (Hauser, 2009).

The high prevalence of multidrug resistance (81.6%) among *P. aeruginosa* isolates reflects patterns commonly reported in tertiary hospitals and ICU settings, where prolonged antibiotic exposure and clonal persistence drive resistance accumulation (Bonomo and Szabo, 2006; Pang et al., 2019). The presence of extensively drug-resistant phenotypes, although limited (4%), is clinically significant because XDR *P. aeruginosa* is strongly associated with treatment failure and increased mortality (Lister et al., 2009). The exoU+ isolates exhibited the highest resistance levels, particularly to β-lactams and carbapenems, consistent with studies linking exoU+ to high-risk hospital clones that frequently co-harbor multiple resistance determinants (Howell et al., 2013; Horna et al., 2019). In contrast, exoS+ isolates showed moderate resistance across cephalosporins and carbapenems, supporting evidence that exoS+ is more often associated with persistence and adaptability rather than extreme resistance phenotypes (Hauser, 2009). Despite genotype-associated trends, the substantial overlap in resistance frequencies across all effector groups indicates heterogeneous resistance profiles, suggesting that resistance evolution is driven more by horizontal gene transfer and local antimicrobial pressure than by virulence genotype alone (Pang et al., 2019). The relatively low resistance to polymyxin B across specimen types aligns with global data identifying polymyxins as last-line agents against MDR *P. aeruginosa*, although emerging resistance underscores the need for cautious use (Poirel et al., 2017). The frequent coexistence of multiple resistance genes within single isolates provides a genetic basis for the observed MDR phenotypes, with exoU+ strains tending to accumulate resistance determinants more consistently than exoT+ or exoY+ isolates, which display more variable gene combinations (Horna et al., 2019).

Regarding the virulence profiles, the results showed that virulence genes were distributed across isolates rather than being limited to a single virulence type, indicating that *P. aeruginosa* uses a combination of virulence factors instead of relying on one dominant mechanism (Hauser, 2009). The exoS+ isolates showed higher frequencies of quorum-sensing and regulatory genes, such as *lasI, rhlI*, and *rhlR*, which are known to support bacterial communication, adaptation, and long-term persistence in the host (Jimenez et al., 2012; Lee and Zhang, 2015). In contrast, exoU+ isolates were more frequently associated with genes involved in tissue damage and cytotoxicity, including *toxA* and *plcH*, which explains their link to severe tissue injury and poor clinical outcomes (Howell et al., 2013). The absence of virulence patterns exclusive to a single type III secretion system genotype supports previous evidence that virulence traits in *P. aeruginosa* are overlapping rather than strictly separated (Lister et al., 2009). Burn and wound isolates showed higher frequencies of genes related to tissue destruction and persistence, reflecting the selective pressure of damaged host tissue (Church et al., 2006). The strong association of exoU+ isolates with serotypes O6 and O11 is consistent with previous reports identifying these serotypes as high-risk lineages frequently linked to severe disease, hospital acquisition, and multidrug resistance. Serotypes O6 and O11 have repeatedly been associated with exoU+ carriage, enhanced cytotoxicity, and poor clinical outcomes, suggesting stable coupling between O-antigen structure and highly virulent genetic backgrounds (Hauser, 2009; Howell et al., 2013). In contrast, the broader serotype distribution observed among exoS+ isolates, including O2, O3, and O5, supports their greater ecological flexibility and ability to circulate across diverse clinical and community settings. The predominance of O6 and O11 among exoT+ isolates further reflects the close linkage between these serotypes and hospital-adapted strains, while the enrichment of O6 and non-typable strains among exoY+ isolates suggests ongoing antigenic variation and immune evasion (Lister et al., 2009).

The results of hierarchical and cluster analyses showed that *P. aeruginosa* isolates have high levels of antibiotic resistance, multiple resistance mechanisms, numerous virulence genes, and diverse serotypes, reflecting strong adaptive capacity and a serious clinical challenge (Lister et al., 2009; Pang et al., 2019). When isolates were grouped by type III secretion system type and sample source, similar trends were observed, with exoU+ isolates carrying the highest resistance and virulence burden, consistent with their association with severe disease and high-risk hospital strains (Hauser, 2009; Howell et al., 2013). However, the wide overlap of resistance and virulence traits across all groups shows that the population is heterogeneous rather than clonal, with no single dominant lineage (Horna et al., 2019). Isolates from the same group did not cluster together, indicating strong diversity even within a single sample type. No fixed combination of resistance genes, virulence factors, or serotypes was conserved, suggesting that each isolate evolved independently under local selective pressures such as antibiotic exposure and host environment rather than through spread of a shared clone (Lister et al., 2009; Pang et al., 2019).

Considering the correlation analysis, Pearson correlation analysis showed that antimicrobial resistance, resistance genes, virulence factors, serotypes, and epidemiological variables were linked by scattered positive and negative associations rather than strong uniform patterns. Positive correlations mainly occurred between related resistance traits, such as fluoroquinolone resistance clustering with other fluoroquinolones and carbapenems, and between MDR and XDR categories, reflecting accumulation of resistance under antibiotic pressure rather than independent events (Lister et al., 2009; Pang et al., 2019). Negative correlations were expected between mutually exclusive groups, including non-MDR versus MDR/XDR and hospital- versus community-acquired isolates, confirming internal consistency of classification. Resistance genes showed weak and inconsistent correlations with phenotypic resistance, indicating that resistance expression is influenced by multiple mechanisms rather than single gene–phenotype relationships (Pang et al., 2019). Of note, the correlation analysis in the present study showed that resistance to the fluoroquinolones (ciprofloxacin and levofloxacin) exhibited a weak to moderate positive association with the exoU+ genotype, whereas weaker or negative correlations were observed with the exoS+ genotype, while exoT+ and exoY+ genotypes showed minimal correlations with fluoroquinolone resistance. This pattern is consistent with previous studies reporting that exoU+ *P. aeruginosa* isolates are more frequently associated with antimicrobial resistance (Horna and Ruiz, 2021; Shaver and Hauser, 2004). In particular, a recent study examining ocular isolates demonstrated a strong association between the exoU+ genotype and resistance to fluoroquinolones and aminoglycosides, while exoS+ isolates were generally more susceptible to these antibiotics (Akter et al., 2025). The stronger association reported in ocular isolates may reflect the selective pressures present in ophthalmic infections, where topical fluoroquinolones and aminoglycosides are commonly used for treatment, promoting the persistence of resistant exoU+ strains. In comparison, the correlations observed in the present study were weaker, which may be attributed to the inclusion of isolates from multiple clinical sources and differences in antimicrobial prescription patterns, potentially reducing the strength of the association between specific T3SS genotypes and antimicrobial resistance.

Isolates recovered from invasive sites such as blood and respiratory samples tended to link with higher levels of multidrug resistance and a broader range of virulence genes than isolates from urine or wound samples. This pattern is consistent with stronger selective pressure in these environments, where intensive antibiotic treatment, invasive procedures, and host immune stress favor survival of more resistant and virulent strains (Lister et al., 2009; Pang et al., 2019). However, these traits were not consistently present in all isolates from the same sample type, indicating substantial variability within each group rather than uniform adaptation. Urine and wound isolates generally carried fewer resistance markers and a smaller set of virulence genes, which aligns with reports that these sites often experience lower antimicrobial pressure compared with bloodstream or respiratory infections, although highly resistant and virulent exceptions are well documented (Hauser, 2009). Certain serotypes appeared more often among blood and respiratory isolates, but their scattered distribution across clusters and resistance profiles shows that serotype alone does not define resistance or virulence behavior. Importantly, no sample type displayed exclusive resistance patterns, virulence gene combinations, or serotype signatures, demonstrating that sample origin influences trait frequency but does not determine isolate identity. Overall, the absence of shared clusters and the wide dispersion of resistance and virulence traits indicate that these trends reflect increased variability rather than clonal expansion or source-driven specialization.

T3SS effector genes showed mixed associations with resistance and virulence traits. The exoU+ displayed weak but consistent positive correlations with MDR status and markers of tissue damage across all specimen types, supporting its role in severe local injury and inflammation (Howell et al., 2013). In contrast, exoS+, exoT+, and exoY+ showed low and inconsistent correlations, suggesting that their distribution is largely independent of resistance burden and clinical severity. Correlation analysis further shows that antimicrobial resistance, resistance genes, virulence factors, T3SS effectors, serotypes, and epidemiological features are largely independently distributed. The observed associations reflect local overlap driven by environmental and clinical pressures rather than shared evolution, supporting a diverse and non-clonal population structure of *P. aeruginosa* (Lister et al., 2009; Horna et al., 2019).

## Conclusion

Hierarchical clustering and intra-group and inter-group analyses showed that isolates clustered in isolate-specific patterns regardless of genotype or infection site, indicating that local selective pressures and independent evolutionary events shape the population structure rather than the spread of dominant clones. *P. aeruginosa* pathogenicity results from flexible combinations of resistance and virulence traits rather than fixed genetic lineages. Despite the high burden of antimicrobial resistance, polymyxin retains clinical value and should be considered a reliable treatment option for severe Pseudomonas aeruginosa infections. The low resistance rate supports its use as a last-line agent in multidrug-resistant cases and highlights the need to preserve its effectiveness through careful stewardship, targeted therapy, and routine susceptibility testing. The findings in this study have direct implications for clinical practice and surveillance. Routine characterization of T3SS effector genotypes, particularly exoU+, can support early risk stratification and help identify infections with a higher likelihood of severe tissue damage. Therefore, incorporating phenotypic, molecular, and clinical data into routine surveillance programs may strengthen early warning systems and improve control of *P. aeruginosa* infections across diverse healthcare environments.

## Data Availability

The original contributions presented in the study are included in the article/**Supplementary Material**. Further inquiries can be directed to the corresponding author.

## Supplementary material

Supplementary Figure 1 Comparative correlation analysis of burn- and eye-derived *Pseudomonas aeruginosa* isolates. **(A)** shows the correlation matrix for burn exudate isolates, and **(B)** shows the correlation matrix for eye exudate isolates. Triangular heatmaps display pairwise correlation coefficients calculated among antimicrobial resistance phenotypes, Variables include resistance to antimicrobials, including non-MDR (non-multidrug resistant), MDR (multidrug resistant), XDR (extensively drug resistant), ATM (aztreonam), TIC (ticarcillin–clavulanate), PIP (piperacillin), C/T (ceftolozane–tazobactam), DOR (doripenem), CAZ (ceftazidime), FDC (cefiderocol), GEN (gentamicin), AMK (amikacin), FEP (cefepime), CIP (ciprofloxacin), LVX (levofloxacin), IMP (imipenem), and MEM (meropenem).Resistance genes, including *bla*_NDM_ (New Delhi metallo-β-lactamase), *bla*_KPC_ (Klebsiella pneumoniae carbapenemase), *bla*_OXA-48_ (oxacillinase-48), *bla*_SHV_ (sulfhydryl variable β-lactamase), *bla*_TEM_ (Temoneira β-lactamase), *bla*_CTX-M_ (cefotaximase), *rmtA* (16S rRNA methyltransferases A), *gyrA* (DNA gyrase subunit A mutation), and *pmrA* (polymyxin resistance regulator). Virulence genes, including *exoS, exoU, exoT*, and *exoY* (type III secretion system effectors), *toxA* (exotoxin A), *aprA* (alkaline protease), *lasB* (elastase), *plcH* (hemolytic phospholipase C), *rhlAB* (rhamnolipid biosynthesis genes), *fliC* (flagellin), *lasI* and *lasR* (quorum sensing regulators), *rhlI* and *rhlR* (quorum sensing regulators), *algD* (alginate biosynthesis), *pslA* and *pelA* (biofilm formation genes), *pvdA* (pyoverdine synthesis), and *pilA* and *pilB* (type IV pili genes). Serotype distribution based on O-antigen, including O1, O2, O3, O5, O6, O11, NT (non-typeable strains) CA (community acquired isolates), HA (Hospital acquired isolates). Clinical sample sources burn, eye, sputum, urine, and wound. Color intensity represents the strength and direction of correlation.

Supplementary Figure 2 **Figure 7** Correlation heatmaps showing associations among antimicrobial resistance, resistance genes, virulence factors, serotypes, and clinical features in *Pseudomonas aeruginosa* isolates from sputum, urine, and wound exudates. **(A)** shows sputum isolates, **(B)** urine isolates, and **(C)** wound isolates. Color intensity represents the strength and direction of correlations, with red indicating positive correlations and blue indicating negative correlations. The dispersed correlation patterns indicate isolate-specific relationships and highlight heterogeneity across specimen types. Triangular heatmaps display pairwise correlation coefficients calculated among antimicrobial resistance phenotypes, Variables include resistance to antimicrobials, including non-MDR (non-multidrug resistant), MDR (multidrug resistant), XDR (extensively drug resistant), ATM (aztreonam), TIC (ticarcillin–clavulanate), PIP (piperacillin), C/T (ceftolozane–tazobactam), DOR (doripenem), CAZ (ceftazidime), FDC (cefiderocol), GEN (gentamicin), AMK (amikacin), FEP (cefepime), CIP (ciprofloxacin), LVX (levofloxacin), IMP (imipenem), and MEM (meropenem).Resistance genes, including *bla*_NDM_ (New Delhi metallo-β-lactamase), *bla*_KPC_ (Klebsiella pneumoniae carbapenemase), *bla*_OXA-48_ (oxacillinase-48), *bla*_SHV_ (sulfhydryl variable β-lactamase), *bla*_TEM_ (Temoneira β-lactamase), *bla*_CTX-M_ (cefotaximase), *rmtA* (16S rRNA methyltransferases A), *gyrA* (DNA gyrase subunit A mutation), and *pmrA* (polymyxin resistance regulator). Virulence genes, including *exoS, exoU, exoT*, and *exoY* (type III secretion system effectors), *toxA* (exotoxin A), *aprA* (alkaline protease), *lasB* (elastase), *plcH* (hemolytic phospholipase C), *rhlAB* (rhamnolipid biosynthesis genes), *fliC* (flagellin), *lasI* and *lasR* (quorum sensing regulators), *rhlI* and *rhlR* (quorum sensing regulators), *algD* (alginate biosynthesis), *pslA* and *pelA* (biofilm formation genes), *pvdA* (pyoverdine synthesis), and *pilA* and *pilB* (type IV pili genes). Serotype distribution based on O-antigen, including O1, O2, O3, O5, O6, O11, NT (non-typeable strains) CA (community acquired isolates), HA (Hospital acquired isolates). Clinical sample sources burn, eye, sputum, urine, and wound. Color intensity represents the strength and direction of correlation.
