# Supplementary figures and images for: Comparative phenotypic and genotypic analysis of distinct Pseudomonas aeruginosa T3SS effector genotypes
Source: Front Cell Infect Microbiol. 2026 Apr 15;16:1792519. doi: 10.3389/fcimb.2026.1792519 (PMC13126654; doi:10.3389/fcimb.2026.1792519)

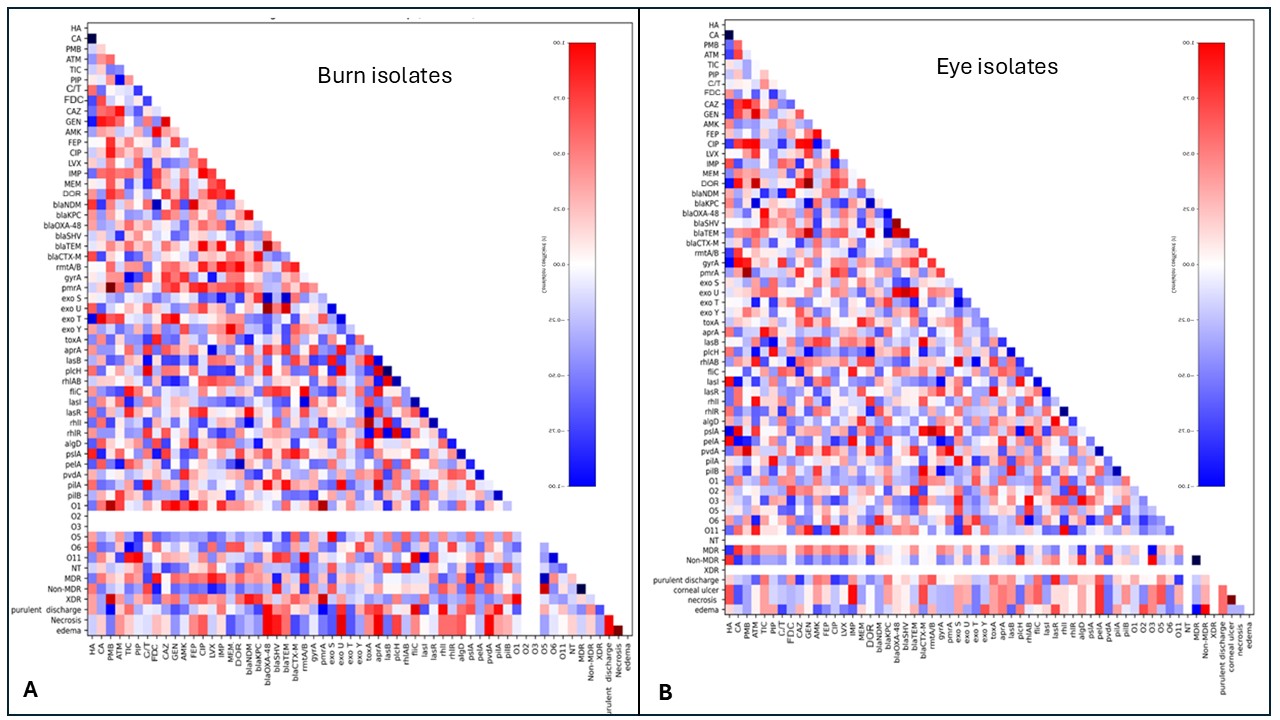

Supplement: Supplementary Figure 1 — Comparative correlation analysis of burn- and eye-derived Pseudomonas aeruginosa isolates. (A) shows the correlation matrix for burn exudate isolates, and (B) shows the correlation matrix for eye exudate isolates. Triangular heatmaps display pairwise correlation coefficients calculated among antimicrobial resistance phenotypes, Variables include resistance to antimicrobials, including non-MDR (non-multidrug resistant), MDR (multidrug resistant), XDR (extensively drug resistant), ATM (aztreonam), TIC (ticarcillin–clavulanate), PIP (piperacillin), C/T (ceftolozane–tazobactam), DOR (doripenem), CAZ (ceftazidime), FDC (cefiderocol), GEN (gentamicin), AMK (amikacin), FEP (cefepime), CIP (ciprofloxacin), LVX (levofloxacin), IMP (imipenem), and MEM (meropenem).Resistance genes, including blaNDM (New Delhi metallo-β-lactamase), blaKPC (Klebsiella pneumoniae carbapenemase), blaOXA-48 (oxacillinase-48), blaSHV (sulfhydryl variable β-lactamase), blaTEM (Temoneira β-lactamase), blaCTX-M (cefotaximase), rmtA (16S rRNA methyltransferases A), gyrA (DNA gyrase subunit A mutation), and pmrA (polymyxin resistance regulator). Virulence genes, including exoS, exoU, exoT, and exoY (type III secretion system effectors), toxA (exotoxin A), aprA (alkaline protease), lasB (elastase), plcH (hemolytic phospholipase C), rhlAB (rhamnolipid biosynthesis genes), fliC (flagellin), lasI and lasR (quorum sensing regulators), rhlI and rhlR (quorum sensing regulators), algD (alginate biosynthesis), pslA and pelA (biofilm formation genes), pvdA (pyoverdine synthesis), and pilA and pilB (type IV pili genes). Serotype distribution based on O-antigen, including O1, O2, O3, O5, O6, O11, NT (non-typeable strains) CA (community acquired isolates), HA (Hospital acquired isolates). Clinical sample sources burn, eye, sputum, urine, and wound. Color intensity represents the strength and direction of correlation. [file Image1.jpeg]

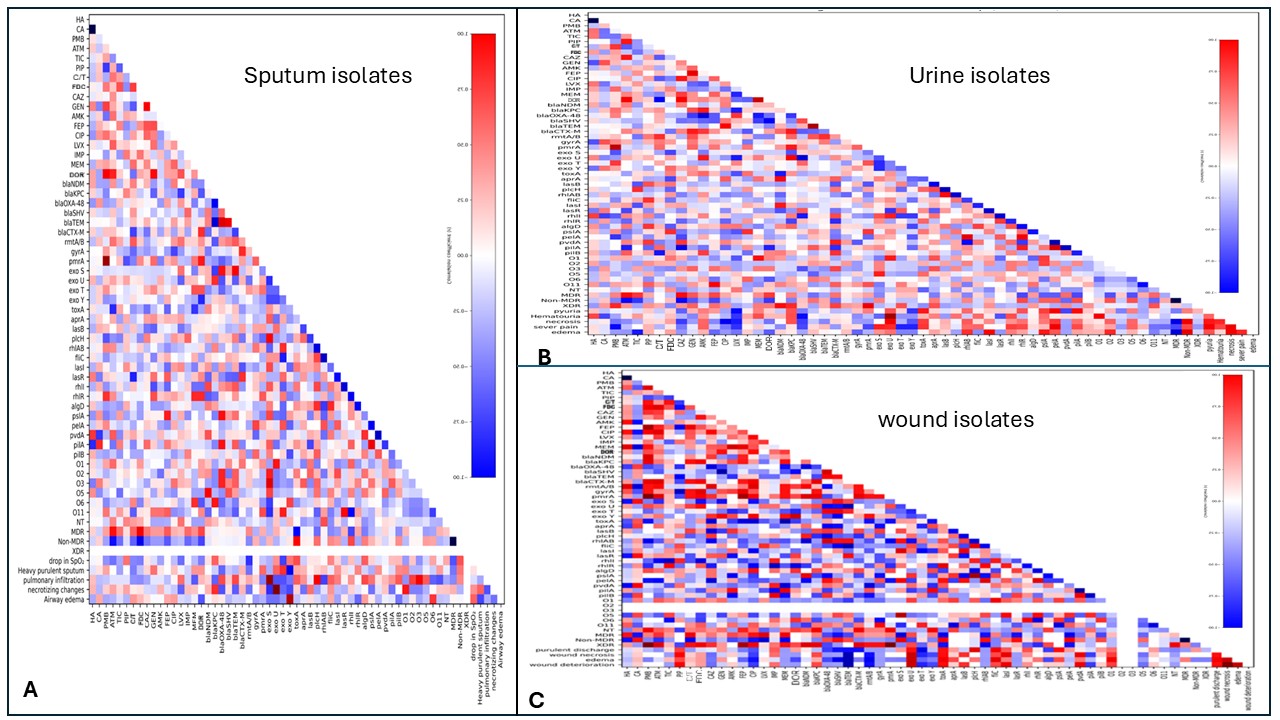

Supplement: Supplementary Figure 2 — Figure 7 Correlation heatmaps showing associations among antimicrobial resistance, resistance genes, virulence factors, serotypes, and clinical features in Pseudomonas aeruginosa isolates from sputum, urine, and wound exudates. (A) shows sputum isolates, (B) urine isolates, and (C) wound isolates. Color intensity represents the strength and direction of correlations, with red indicating positive correlations and blue indicating negative correlations. The dispersed correlation patterns indicate isolate-specific relationships and highlight heterogeneity across specimen types. Triangular heatmaps display pairwise correlation coefficients calculated among antimicrobial resistance phenotypes, Variables include resistance to antimicrobials, including non-MDR (non-multidrug resistant), MDR (multidrug resistant), XDR (extensively drug resistant), ATM (aztreonam), TIC (ticarcillin–clavulanate), PIP (piperacillin), C/T (ceftolozane–tazobactam), DOR (doripenem), CAZ (ceftazidime), FDC (cefiderocol), GEN (gentamicin), AMK (amikacin), FEP (cefepime), CIP (ciprofloxacin), LVX (levofloxacin), IMP (imipenem), and MEM (meropenem).Resistance genes, including blaNDM (New Delhi metallo-β-lactamase), blaKPC (Klebsiella pneumoniae carbapenemase), blaOXA-48 (oxacillinase-48), blaSHV (sulfhydryl variable β-lactamase), blaTEM (Temoneira β-lactamase), blaCTX-M (cefotaximase), rmtA (16S rRNA methyltransferases A), gyrA (DNA gyrase subunit A mutation), and pmrA (polymyxin resistance regulator). Virulence genes, including exoS, exoU, exoT, and exoY (type III secretion system effectors), toxA (exotoxin A), aprA (alkaline protease), lasB (elastase), plcH (hemolytic phospholipase C), rhlAB (rhamnolipid biosynthesis genes), fliC (flagellin), lasI and lasR (quorum sensing regulators), rhlI and rhlR (quorum sensing regulators), algD (alginate biosynthesis), pslA and pelA (biofilm formation genes), pvdA (pyoverdine synthesis), and pilA and pilB (type IV pili genes). Serotype distribution based on O-an [file Image2.jpeg]
