# Supplementary material for: Comparative phenotypic and genotypic analysis of distinct Pseudomonas aeruginosa T3SS effector genotypes
Source: Front Cell Infect Microbiol. 2026 Apr 15;16:1792519. doi: 10.3389/fcimb.2026.1792519 (PMC13126654; doi:10.3389/fcimb.2026.1792519)
Supplement: Supplementary file 3 [file Table1.docx]

**Table S1: Primer sequences and amplification size of type III secretion system exo genes (exoS, exoT, exoU, and exoY), virulence and resistance genes in *Pseudomonas aeruginosa***

| **Target gene** | **Primer sequences (5' - 3')** | **Amplicon size (bp)** | **References** |
| --- | --- | --- | --- |
| *oprL* | F: ATGGAAATGCTGAAATTCGGC′  R: CTTCTTCAGCTCGACGCGACG |  | **Spilker et al. 2004** |
| *exo S* | F: GCG AGG TCA GCA GAG TAT CG R: TTC GGC GTC ACT GTG GAT GC | 118 | **Jarjees, 2020 ;**  **Elnagar et al., 2022** |
| *exo T* | F: AAT CGC CGT CCA ACT GCA TGC G R: TGT TCG CCG AGG TAC TGC TC | 152 |  |
| *exo U* | F: CCG TTG TGG TGC CGT TGA AG R: CCA GAT GTT CAC CGA CTC GC | 134 |  |
| *exo Y* | FW: CGG ATT CTA TGG CAG GGA GG RV: GCC CTT GAT GCA CTC GAC CA | 289 |  |
| **Virulence genes** | | | |
| *toxA* | **F:** GGAGCGCAACTATCCCACT  **R:** TGGTAGCCGACGAACACATA | 150 | **Sabharwal et al., 2014** |
| *aprA* | **F:** GTCGACCAGGCGGCGGAGCAGATA  **R:** GCCGAGGCCGCCGTAGAGGATGTC | 993 | **Sabharwal et al., 2014** |
| *lasB* | **F:** TTCTACCCGAAGGACTGATAC  **R:** AACACCCATGATCGCAAC | 153 | **Sabharwal et al., 2014** |
| *plcH* | **F:** GAAGCCATGGGCTACTTCAA  **R:** AGAGTGACGAGGAGCGGTAG | 307 | **Sabharwal et al., 2014** |
| *rhlAB* | **F:** TCATGGAATTGTCACAACCGC  **R:** ATACGGCAAAATCATGGCAAC | 151 | **Sabharwal et al., 2014** |
| *fliC* | **F:** GGCAGCTGGTTNGCCTG  **R:** GGCCTGCAGATCNCCAA | 1250 | **Sabharwal et al., 2014** |
| *lasI* | **F:** CGTGCTCAAGTGTTCAAGG  **R:** TACAGTCGGAAAAGCCCAG | 295 | **Sabharwal et al., 2014** |
| *lasR* | **F:** AAGTGGAAAATTGGAGTGGAG  **R:** GTAGTTGCCGACGACGATGAAG | 130 | **Sabharwal et al., 2014** |
| *rhlI* | **F:** TTCATCCTCCTTTAGTCTTCCC  **R:** TTCCAGCGATTCAGAGAGC | 155 | **Sabharwal et al., 2014** |
| *rhlR* | **F:** TGCATTTTATCGATCAGGGC  **R:** CACTTCCTTTTCCAGGACG | 133 | **Sabharwal et al., 2014** |
| *algD* | **F:** CGAGAAGTCCGAACGCCACAC  **R:** ATCGGCGGGAAGTCGTA | 186 | **Wang et al., 2025** |
| *pslA* | **F:** GGCCTGTTTCCCTACCT  **R:** GCGGATGTCGTGGTTG | 207 | **Wang et al., 2025** |
| *pelA* | **F:** GGCCTGCTCGAATACCTC  **R:** TGACCTTGAGTTTCTGCGACA | 265 | **Wang et al., 2025** |
| *pvdA* | **F:** GACTCAGGCAACTGCAAC  **R:** TTCAGGTGCTGGTACAGG | 1281 | **Fazeli and Momtaz, 2014** |
| *pilA* | **F:** ACAGCATCCAACTGAGCG  **R:** TTGACTTCCTCCAGGCTG | 1675 | **Fazeli and Momtaz, 2014** |
| *pilB* | **F:** TCGAACTGATGATCGTGG  **R:** CTTTCGGAGTG*AACA*TCG | 408 | **Fazeli and Momtaz, 2014** |
| **Resistance genes** | | | |
| *bla*_NDM_ | F: TAAAATACCTTGAGCGGGC  R: AAATGGAAACTGGCGACC | 439 | **Mlynarcik etal., 2016** |
| *bla*_KPC_ | F: TGTTGCTGAAGGAGTTGGGC  R: ACGACGGCATAGTCATTTGC | 340 | **Mlynarcik etal., 2016** |
| *bla*_OXA-48_ | F: GCTTGATCGCCCTCGATT  R: GATTTGCTCCGTGGCCGAAA | 281 | **Gurung etal., 2020** |
| *bla_S_*_HV_ | F: AAAGATCCACTATCGCCAGCAG R: ATTCAGTTCCGTTTCCCAGCGG | 231 | **Mohanam, and Menon, 2022** |
| *bla*_TEM_ | F: CATTTCCGTGTCGCCCTTATTC R: CGTTCATCCATAGTTGCCTGAC | 800 | **Mohanam, and Menon, 2022** |
| *bla*_CTX-M_ | AATCACTGCGCCAGTTCACGCT  GAACGTTTCGTCTCCCAGCTGT | 479 | **Chia etal., 2005** |
| *rmtA/B* | F: AGGTTGTTTCCATTTCTGAG  R: TCTCTTCCATTCCCTTCTCC | 590 | **Yamane et al., 2005** |
| *gyrA* | F: GTGTGCTTTATGCCATGAG  R: GGTTTCCTTTTCCAGGTC | 287 | **Nguyen et al., 2018** |
| *pmrA* | F: GACCAAGCCCTTCGATCTC  R: AGGTGGTGGACGTGGACTT | 294 | **Jafari-Ramedani et al., 2024** |
